# Investigation of the shared molecular mechanisms and hub genes between myocardial infarction and depression

**DOI:** 10.3389/fcvm.2023.1203168

**Published:** 2023-07-21

**Authors:** Mengxi Wang, Liying Cheng, Ziwei Gao, Jianghong Li, Yuhan Ding, Ruijie Shi, Qian Xiang, Xiaohu Chen

**Affiliations:** ^1^ Department of Cardiology, Affiliated Hospital of Nanjing University of Chinese Medicine, Nanjing, China; ^2^ Department of Cardiology, Jiangsu Province Hospital of Chinese Medicine, Nanjing, China; ^3^ First Clinical Medical College, Nanjing University of Chinese Medicine, Nanjing, China; ^4^ State Key Laboratory of Component-Based Chinese Medicine, Tianjin University of Traditional Chinese Medicine, Tianjin, China

**Keywords:** myocardial infarction, depression, immune inflammation, pathogenesis, diagnosis, molecular subtype

## Abstract

**Background:**

The pathogenesis of myocardial infarction complicating depression is still not fully understood. Bioinformatics is an effective method to study the shared pathogenesis of multiple diseases and has important application value in myocardial infarction complicating depression.

**Methods:**

The differentially expressed genes (DEGs) between control group and myocardial infarction group (M-DEGs), control group and depression group (D-DEGs) were identified in the training set. M-DEGs and D-DEGs were intersected to obtain DEGs shared by the two diseases (S-DEGs). The GO, KEGG, GSEA and correlation analysis were conducted to analyze the function of DEGs. The biological function differences of myocardial infarction and depression were analyzed by GSVA and immune cell infiltration analysis. Four machine learning methods, nomogram, ROC analysis, calibration curve and decision curve were conducted to identify hub S-DEGs and predict depression risk. The unsupervised cluster analysis was constructed to identify myocardial infarction molecular subtype clusters based on hub S-DEGs. Finally, the value of these genes was verified in the validation set, and blood samples were collected for RT-qPCR experiments to further verify the changes in expression levels of these genes in myocardial infarction and depression.

**Results:**

A total of 803 M-DEGs, 214 D-DEGs, 13 S-DEGs and 6 hub S-DEGs (CD24, CSTA, EXTL3, RPS7, SLC25A5 and ZMAT3) were obtained in the training set and they were all involved in immune inflammatory response. The GSVA and immune cell infiltration analysis results also suggested that immune inflammation may be the shared pathogenesis of myocardial infarction and depression. The diagnostic models based on 6 hub S-DEGs found that these genes showed satisfactory combined diagnostic performance for depression. Then, two molecular subtypes clusters of myocardial infarction were identified, many differences in immune inflammation related-biological functions were found between them, and the hub S-DEGs had satisfactory molecular subtypes identification performance. Finally, the analysis results of the validation set further confirmed the value of these hub genes, and the RT-qPCR results of blood samples further confirmed the expression levels of these hub genes in myocardial infarction and depression.

**Conclusion:**

Immune inflammation may be the shared pathogenesis of myocardial infarction and depression. Meanwhile, hub S-DEGs may be potential biomarkers for the diagnosis and molecular subtype identification of myocardial infarction and depression.

## 1. Introduction

Myocardial infarction, the most serious type of coronary heart disease, is a kind of disease with clinical evidence of myocardial injury and ischemia (1). Myocardial infarction is one of the most dangerous diseases in the world with high morbidity, high disability rate and high fatality rate, which has brought heavy burden to society (2). Depression is a kind of mental disorder disease, mainly manifested by various negative emotions, including guilt, sadness and so on (3). In addition, it is often accompanied by sleep disorders, fatigue, loss of appetite, slow thinking and other symptoms. With the continuous development of social economy, the incidence of depression has gradually increased due to the accelerated pace of life and the increase of pressure, mental health has become an important public health problem (4).

In recent years, an increasing number of evidence has shown a strong link between myocardial infarction and depression. On the one hand, myocardial infarction is one of the important risk factors for depression. A systematic review showed that acute myocardial infarction was followed by depression in 28.7 percent of patients (5). On the other hand, depression also increases the risk of myocardial infarction. The study found that depression increased the risk of myocardial infarction in patients with stable angina by about 31 percent (6). In addition, depression has a significant negative impact on the prognosis of patients with myocardial infarction, whether the depression occurred before or after the myocardial infarction. Some studies have shown that patients with depression after myocardial infarction have significantly higher rates of mortality and cardiovascular adverse events within 16 months, and significantly higher rates of cardiac mortality within 5 years (7, 8). Another study found that patients who had been diagnosed with depression prior to myocardial infarction had a 7% increased risk of death within 1 year and 9% increased risk of death within 19 years compared with patients without depression (9). Thus, it can be seen that myocardial infarction and depression could interact with each other, leading to a worsening of the disease process and serious damage to human health. However, at present, the pathogenesis of myocardial infarction complicating depression is still unclear, effective diagnosis and treatment methods are still lacking (10, 11). Therefore, it is of great clinical significance to explore the pathogenesis of myocardial infarction complicating depression, search for early diagnosis biomarkers and targeted therapeutic measures.

**Figure 1:**
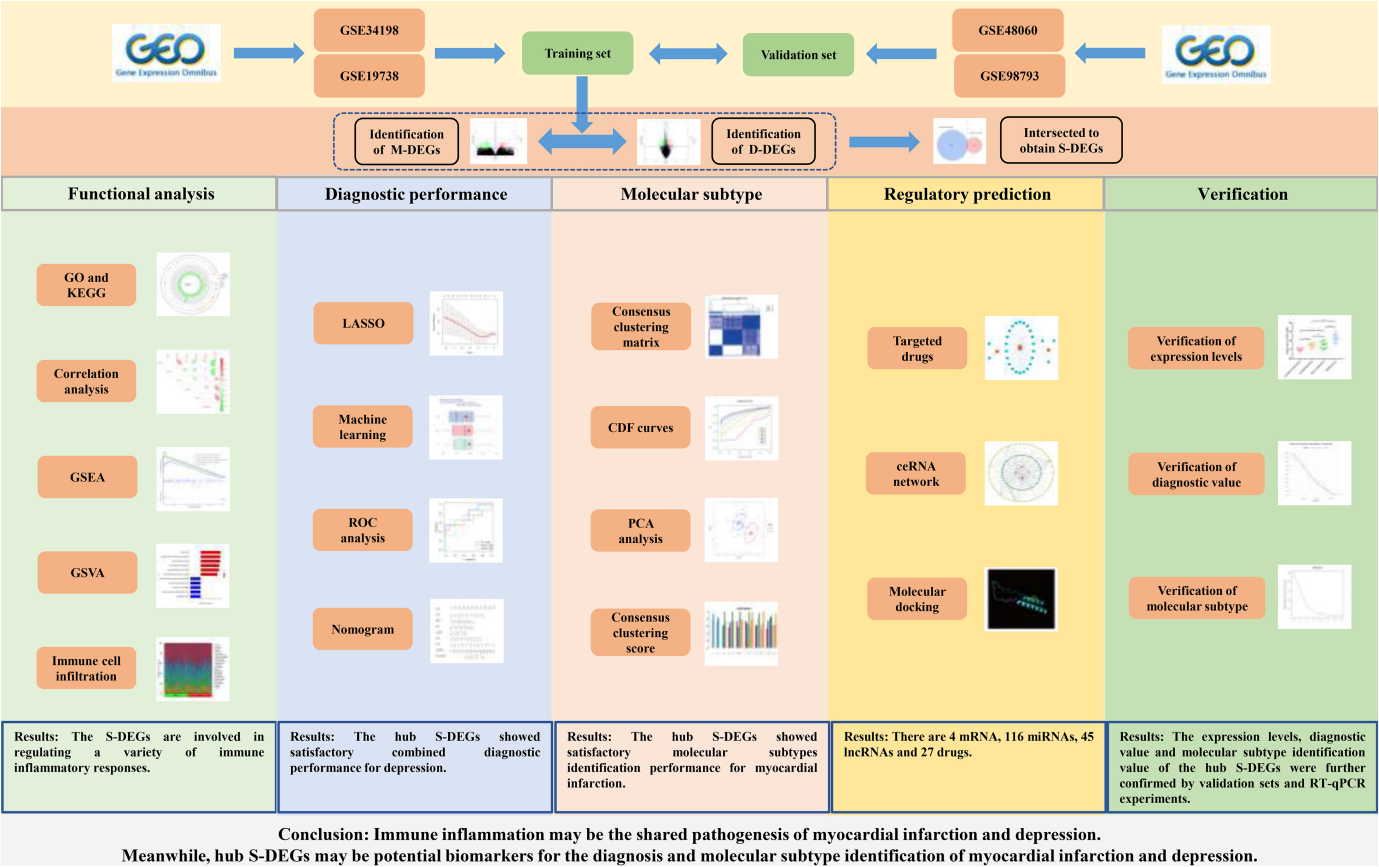
The flowchart of this study.

Bioinformatics analysis could integrate experimental data from multiple sources and analyze the data from multiple levels and angles, which has great advantages in studying the pathogenesis of diseases, exploring diagnostic markers and therapeutic measures. However, there are no studies that apply this technique to myocardial infarction and depression. Therefore, this study attempted to explore the pathogenesis of myocardial infarction complicating depression from the gene level through bioinformatics analysis combined with experimental verification, and to find out the genes with diagnostic value and potential therapeutic measures, in order to provide valuable references for the research of myocardial infarction and depression. The workflow of this study is presented in the Figure 1.

## 2. Material and methods

### 2.1. Data acquisition

We used the keywords “myocardial infarction”, “depression” or “depressive disorder” to search the myocardial infarction datasets and depression datasets in the Gene Expression Omnibus (GEO) database. The retrieved results were then filtered according to the following criteria: (1) The species studied was Homo sapiens. (2) The disease group was myocardial infarction or depression patients, and the control group was healthy people. (3) The test sample was whole blood. Finally, we selected two myocardial infarction datasets and two depression datasets with the largest sample size in the results as the training and validation sets respectively. The GSE34198 dataset was defined as the training set for myocardial infarction, containing 49 patients with myocardial infarction and 48 healthy control population. The GSE19738 dataset was defined as the training set for depression, containing 33 patients with depression and 34 healthy control population. The GSE48060 dataset was defined as the validation set for myocardial infarction, containing 31 patients with myocardial infarction and 21 healthy control population. The GSE98793 dataset was defined as the validation set for depression, containing 128 patients with depression and 64 healthy control population. The details of the 4 GEO datasets were shown in the Supplementary Table S1.

### 2.2. Quality control

For the datasets containing “cel files” in the original data, we evaluated their quality with the R packages “affy”, “affyPLM” and “RColorBrewer” using three analysis methods: relative log expression (RLE), normalized unscaled standard errors (NUSE) and RNA degradation curve. For datasets that do not contain “cel files” in the original data, we searched the original literature corresponding to these datasets. If the dataset result was verified by RT-qPCR experiment in the original literature, its quality was considered to meet the requirement.

### 2.3. Differential expression analysis

The log2 conversion was used to correct the data before the differential analysis was performed. Next, the R package “limma” was used to identify differential expression genes (DEGs) between myocardial infarction group and control group (M-DEGs), or between depression group and control group (D-DEGs). The *P*-value <0.05 and |log2 fold change| >0.2 was considered be significant difference. Then, M-DEGs and D-DEGs were intersected to obtain the differential expression genes shared by the two diseases (S-DEGs). After that, the heatmap of DEGs was drawn with “pheatmap” package, and the volcano plot of DEGs was drawn with “ggplot2” package. Finally, the R package “RCircos” was used to visualize the position of S-DEGs on the chromosome.

### 2.4. Unsupervised clustering for myocardial infarction patients based on S-DEGs

The unsupervised clustering analysis of myocardial infarction patients was performed using “ConsensusClusterPlus” package based on S-DEGs. The optimal number of molecular subtype clusters was evaluated comprehensively according to consensus clustering matrix, cumulative distribution function (CDF) curves, CDF delta area curves and consensus clustering score.

### 2.5. Gene set variation analysis (GSVA)

The R package “GSVA” was used for GSVA analysis with Gene Ontology (GO) and Kyoto Encyclopedia of Genes and Genomes (KEGG) data files as references. The R package “limma” was used to identify differential signaling pathways and biological functions between disease group and control group, or between different molecular subtype clusters. The absolute *t* value of GSVA score more than 2 was considered as a significant difference.

### 2.6. Single-gene gene set enrichment analysis (GSEA)

Single-gene GSEA was performed on the S-DEGs in this study to explore the biological functions and signaling pathways associated with these genes. The specific analysis steps are as follows, calculating the correlation between all other genes and these key genes in the entire dataset and sequencing the gene sets according to the correlation. With the GO and KEGG data files as reference, the biological functions and signaling pathways enrichment of each gene set were evaluated to find the significantly enriched biological functions and signaling pathways.

### 2.7. Immune cell infiltration, protein–protein interaction (PPI) network, GO and KEGG analysis

The methods of immune cell infiltration analysis refer to previous studies (12, 13). Based on the CIBERSORT algorithm, the relative abundance and proportion of 22 types of immune cells in each sample were evaluated to explore differences in immune cell infiltration between the control group and myocardial infarction group or depression group, as well as between clusters of different molecular subtypes. The STRING database was used to carry out PPI network analysis of S-DEGs. The R package “clusterProfiler” and “circlize” were used to perform GO and KEGG analysis of M-DEGs, D-DEGs and S-DEGs.

### 2.8. Construction and evaluation of diagnostic model and molecular subtype identification model

Based on the methods of previous studies, hub genes for depression diagnosis were selected (14, 15). Four machine learning methods, including least absolute shrinkage and selection operator (LASSO) regression, random forest model (RF), support vector machine model (SVM), generalized linear model (GLM) were performed to evaluate the value of S-DEGs in diagnosing depression. The intersection of the top 10 S-DEGs with diagnostic value selected by these 4 methods was performed to obtain the hub S-DEGs. After that, R packages “caret” was used to construct 3 machine learning models for diagnosis and molecular subtypes identification based on the hub S-DEGs. Finally, the diagnosis and identification value of the 3 machine learning models was evaluated by cumulative residual distribution curves, residual boxplots and receiver operating characteristic (ROC) curves.

### 2.9. Establishment and verification of the nomogram

The R package “rms” was used to build the nomogram based on 6 hub S-DEGs. The different scores were assigned according to the expression level of each gene and the sum of all gene scores could reflect the occurrence probability of disease. Calibration curves and decision curve analysis (DCA) were used to evaluate the predictive power of the nomogram.

### 2.10. Verification of the diagnostic value and molecular subtype identification value of hub S-DEGs in the validation set

In order to further verify the diagnostic value of hub S-DEGs for depression and the molecular subtype identification value for myocardial infarction, we selected the dataset GSE98793 as the depression validation set and the dataset GSE48060 as the myocardial infarction validation set. In the depression validation set, we assessed whether the expression levels of hub S-DEGs were consistent with those in the training set, and evaluated whether the diagnostic performance of hub S-DEGs was as satisfactory as they were in the training set through machine learning and ROC analysis. In the myocardial infarction validation set, unsupervised clustering analysis, PCA analysis, immune cell infiltration analysis, machine learning, and ROC analysis were used to evaluate whether the hub S-DEGs could identify molecular subtypes similar to the training set and whether they could show the same satisfactory molecular subtype identification performance.

### 2.11. RT-qPCR

To verify expression levels of S-DEGs in peripheral blood of patients with myocardial infarction, depression, and myocardial infarction complicating depression, we collected 4 groups of patients: patients without myocardial infarction or depression (*n* = 10), patients with myocardial infarction but without depression (*n* = 10), patients with depression but without myocardial infarction (*n* = 10), myocardial infarction complicating depression patients (*n* = 10). Baseline characteristics of these patients were presented in the Supplementary Table S2. Peripheral blood RNA of these patients was extracted by TRIzol method for RT-qPCR detection. The RT-qPCR experiment followed the following steps and parameters recommended by the instruction. The reverse transcription program was set as: 25°C for 5 min, 55°C for 15 min, 85°C for 5 min. The amplification procedure was set as: pre-denaturation for 5 min at 95°C, followed by 40 cycles of 95°C for 10 s and 60°C for 30 s. The mRNA expression level was calculated by △△Ct method with GAPDH as internal reference. The primer sequences are shown in Supplementary Table S3. The Primer synthesis was completed by Generay Biotech. This study was approved by the Ethics Committee of Jiangsu Province Hospital of Chinese Medicine (Ethical Approval Number: 2018NL-105-04).

### 2.12. Construction the ceRNA network of mRNA-miRNA-lncRNA interactions

The miRanda, miRDB and TargetScan databases were used to predict miRNAs that could target the hub S-DEGs, and the lncBase and mircode databases were used to predict lncRNAs that could target these miRNAs. The Cytoscape software was used to construct the ceRNA network of mRNA-miRNA-lncRNA interactions.

### 2.13. Prediction of genes-targeted drugs

The DSigDB database was used to predict potential drugs targeting hub S-DEGs, and the Cytoscape software was used to visualize the predicted results.

### 2.14. Molecular docking

The 2D structures of targeted drugs were downloaded from the PubChem database. Chem3D was used to convert 2D structures into 3D structures. The 3D structures of hub S-DEGs were downloaded from the PDB database or UniProt database. Autodock Tools was used for active pocket location. Finally, Autodock Vina was employed for molecular docking to predict the binding sites and binding free energy between the targeted drugs and hub S-DEGs. The lower binding free energy is, the higher binding strength will be.

### 2.15. Statistical analysis

The software of R 4.2.1 was used for bioinformatics analysis and figures drawing. The software of GraphPad Prism 7.0 was used for RT-qPCR data analysis. One-way ANOVA was performed to compare three or more groups of data. The *P* value <0.05 was considered statistically significant.

## 3. Results

### 3.1. Identification and enrichment analysis of M-DEGs and D-DEGs

**Figure 2:**
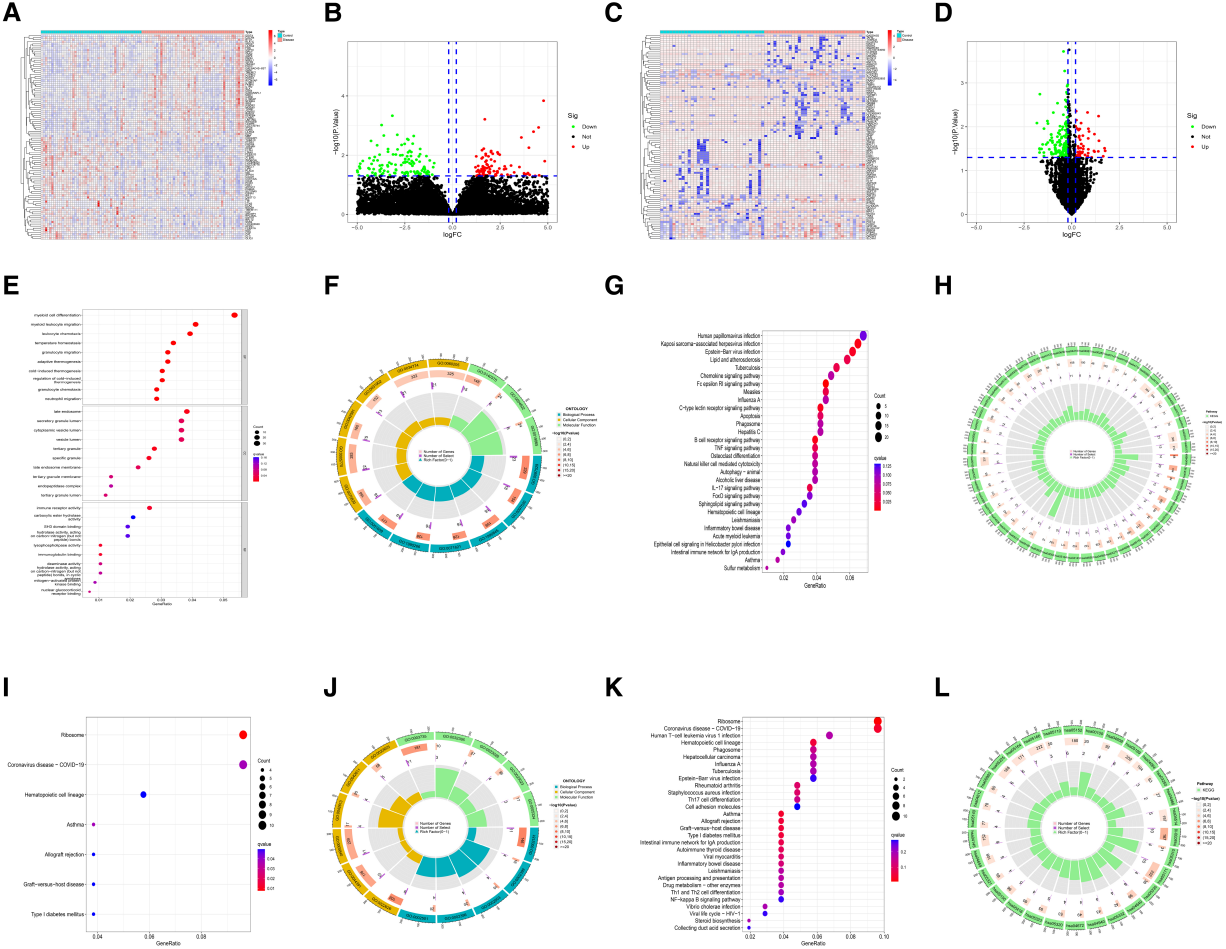
Identification and functional analysis of M-DEGs and D-DEGs. (**A**) The heatmap of M-DEGs. (**B**) The volcano plot of M-DEGs. (**C**) The heatmap of D-DEGs. (**D**) The volcano plot of D-DEGs. (**E**) The bubble chart of GO analysis of M-DEGs. (**F**) The circle diagram of GO analysis of M-DEGs. (**G**) The bubble chart of KEGG analysis of M-DEGs. (**H**) The circle diagram of KEGG analysis of M-DEGs. (**I**) The bubble chart of GO analysis of D-DEGs. (**J**) The circle diagram of GO analysis of D-DEGs. (**K**) The bubble chart of KEGG analysis of D-DEGs. (**L**) The circle diagram of KEGG analysis of D-DEGs.

The quality of the four datasets included in this study met the requirement. The results of the datasets GSE34198 and GSE19738 were verified by RT-qPCR experiment in the original literature (16, 17). The quality of datasets GSE48060 and GSE98793 was evaluated using three analysis methods (RLE, NUSE and RNA degradation curve), and the evaluation results were shown in Supplementary Figures S1A–F. In the myocardial infarction and depression training sets, 803 M-DEGs and 214 D-DEGs were identified respectively (Figures 2A–D). The GO and KEGG enrichment analysis of 803 M-DEGs showed that these genes were mainly involved in multiple cellular or cytokine-mediated immune inflammatory responses, such as leukocyte chemotaxis, immune receptor activation, chemokine signaling pathway, B cell receptor signaling pathway, TNF signaling pathway and IL-17 signaling pathway (Figures 2E–H). Then, GO and KEGG enrichment analysis were performed on 214 D-DEGs, and it was also found that these genes could regulate various immune inflammatory reactions, such as Th17 cell differentiation, Th1 cell differentiation, Th2 cell differentiation and NF-κB signaling pathway (Figures 2I–L). These results suggest that immune inflammation may be the shared pathogenesis of myocardial infarction and depression.

### 3.2. Identification of shared pathogenesis of myocardial infarction and depression

**Figure 3:**
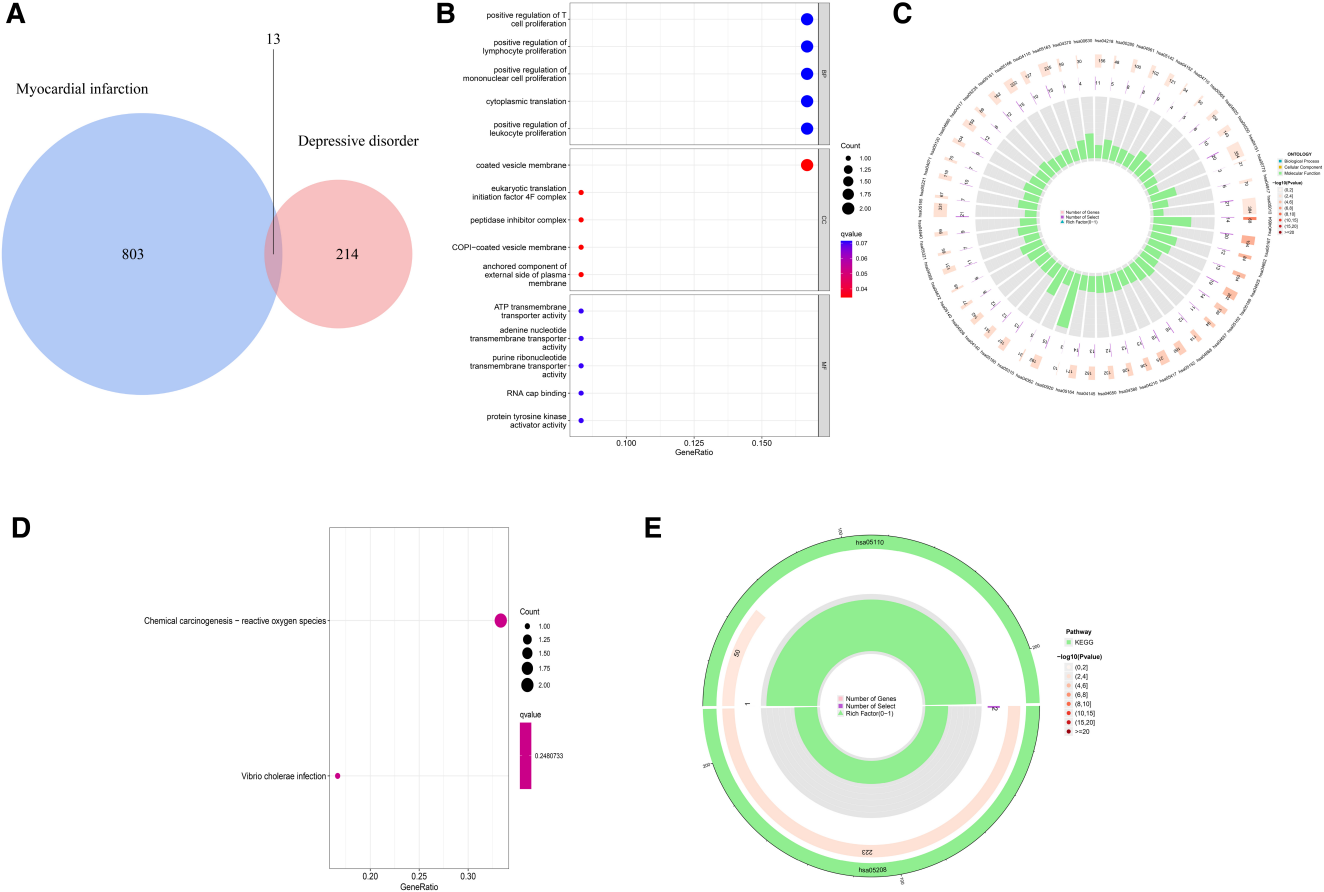
Identification and functional analysis of S-DEGs. (**A**) The S-DEGs were identified by Venn diagram. (**B**) The bubble chart of GO analysis of S-DEGs. (**C**) The circle diagram of GO analysis of S-DEGs. (**D**) The bubble chart of KEGG analysis of S-DEGs. (**E**) The circle diagram of KEGG analysis of S-DEGs.

**Figure 4:**
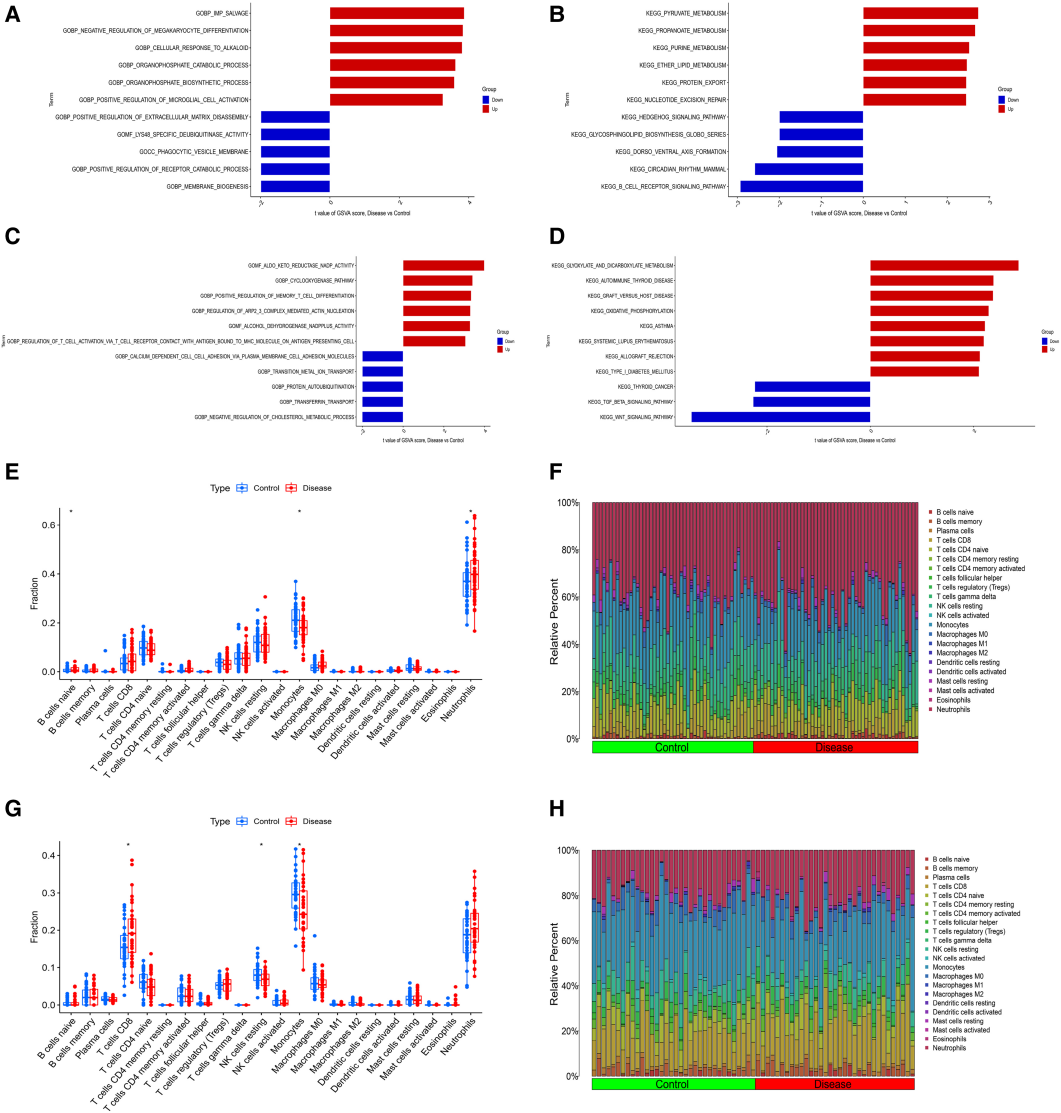
Differential function analysis between control group and myocardial infarction group or depression group. (**A**) The results of GSVA analysis showing the differences in biological functions between myocardial infarction group and control group (red bars represent activation of these biological functions in the myocardial infarction group, blue bars represent inhibition of these biological functions in the myocardial infarction group). (**B**) The results of GSVA analysis showing the differences in signaling pathways between myocardial infarction group and control group (red bars represent activation of these signal pathways in the myocardial infarction group, blue bars represent inhibition of these signal pathways in the myocardial infarction group). (**C**) The results of GSVA analysis showing the differences in biological functions between depression group and control group. (**D**) The results of GSVA analysis showing the differences in signaling pathways between depression group and control group. (**E**) The box plot showing the differences in infiltrated immune cells between myocardial infarction group and control group. (**F**) The bar plot showing relative proportion of 22 infiltrated immune cells in myocardial infarction group and control group. (**G**) The box plot showing the differences in infiltrated immune cells between depression group and control group. (**H**) The bar plot showing relative proportion of 22 infiltrated immune cells in depression group and control group.

In order to explore the shared pathogenesis of myocardial infarction and depression, 13 S-DEGs were obtained by the intersection of 803 M-DEGs and 214 D-DEGs (Figure 3A). Then, GO and KEGG enrichment analysis were performed on S-DEGs, the results showed that these genes were also involved in a variety of immune inflammatory responses, including T cell proliferation and activation, lymphocyte proliferation and monocyte proliferation (Figures 3B–E). To further investigate the role of immune inflammation in myocardial infarction and depression, GSVA analysis and immune cell infiltration analysis were performed on the training set of myocardial infarction and depression. The results of GSVA analysis showed significant changes in B cell receptor signaling pathways in patients with myocardial infarction (Figures 4A,B), and significant changes in T cell differentiation and activation in patients with depression (Figures 4C,D). The results of immune cell infiltration analysis showed that there were significant differences in the infiltration levels of naive B cell, monocyte and neutrophil in patients with myocardial infarction (Figures 4E,F), and significant differences in the infiltration levels of CD8^+^ T cell, natural killer cell and monocyte in patients with depression (Figures 4G,H). These results further suggest that immune inflammation may be the shared pathogenesis of myocardial infarction and depression.

### 3.3. Construction of diagnostic model based on hub S-DEGs

**Figure 5:**
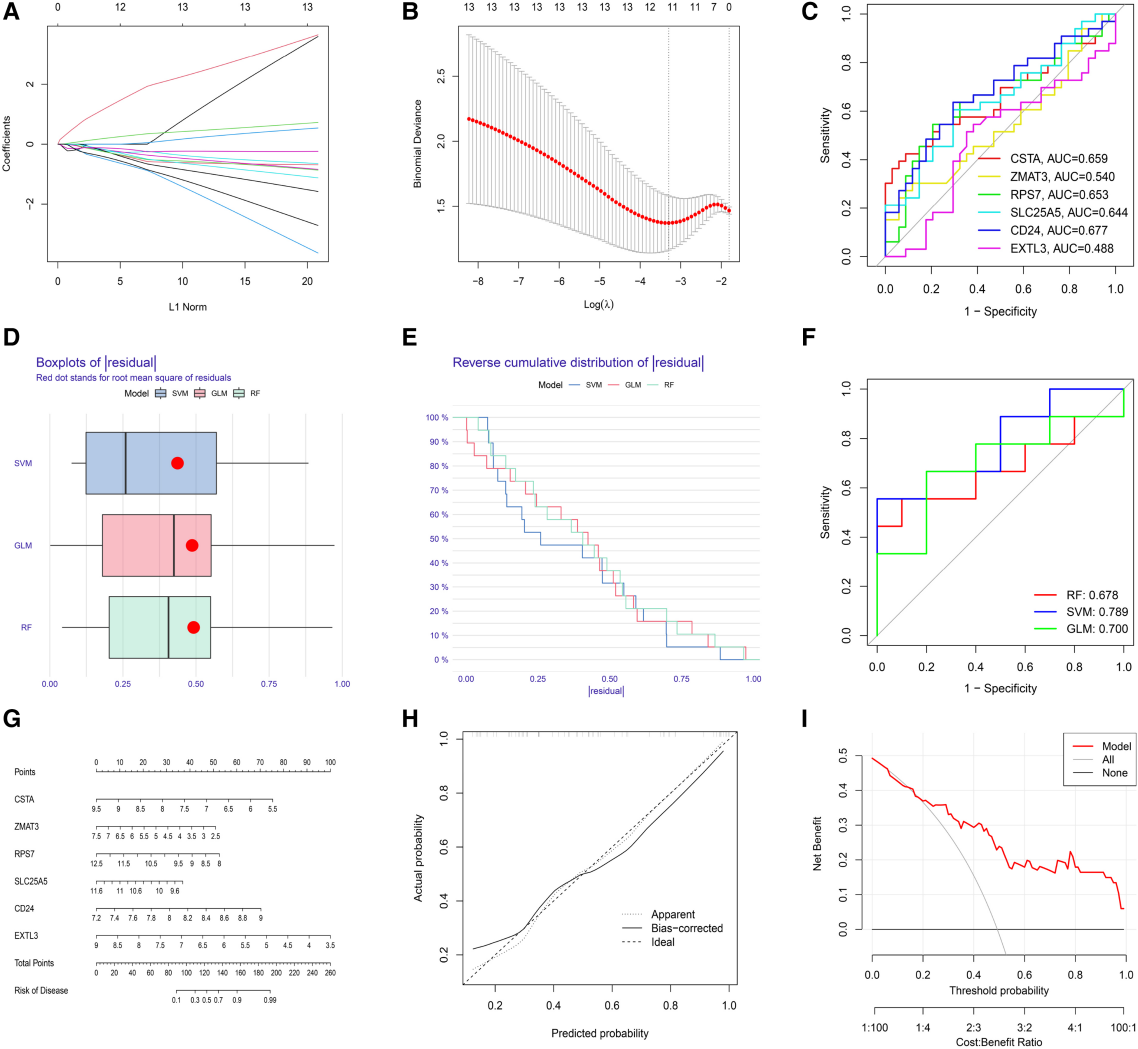
Evaluation of the diagnostic value of S-DEGs. (**A**) The LASSO coefficient of 13 S-DEGs of the diagnostic value. (**B**) The optimal lambda value was selected by LASSO regression based on cross-validation. (**C**) Evaluation of the diagnostic value of 6 hub S-DEGs individually by ROC analysis. (**D**) The residual boxplots of 3 machine learning models. (**E**) The cumulative residual distribution curves of 3 machine learning models. (**F**) The ROC curves of 3 machine learning models. (**G**) The nomogram for predicting the risk of depression based on 6 hub S-DEGs. (**H**) The calibration curves of the nomogram. (**I**) The DCA of the nomogram.

The LASSO regression was performed to evaluate the value of 13 S-DEGs in the diagnosis of depression and 11 genes with the best diagnostic value were selected (Figures 5A,B). Then, other 3 machine learning methods were used to pick out the top 10 genes for diagnostic value respectively. Through the intersection of genes selected by 4 machine learning methods, 6 hub S-DEGs were obtained (CD24, CSTA, EXTL3, RPS7, SLC25A5 and ZMAT3). The PPI network analysis result showed no interaction between these genes (Supplementary Figure S2). Next, we evaluated the diagnostic value of the 6 hub S-DEGs individually, and the results showed that all the individual genes had low diagnostic efficacy (Figure 5C). After that, we tried to improve the diagnostic efficiency by constructing multi-gene association diagnostic models. We constructed 3 machine learning models based on the 6 hub S-DEGs, including RF, SVM and GLM. Three methods were used to select the best diagnostic model (cumulative residual distribution curves, residual boxplots and ROC curves). The results found that the SVM model showed the best diagnostic efficiency (area under ROC curve was 0.789). The machine learning model construction results were shown in Figures 5D–F. Finally, in order to predict the risk of disease more accurately, we constructed the nomogram based on 6 hub S-DEGs. The results of calibration curves and DCA showed that the prediction performance of the nomogram is satisfactory (Figures 5G–I).

### 3.4. Functional analysis of the hub S-DEGs

**Figure 6:**
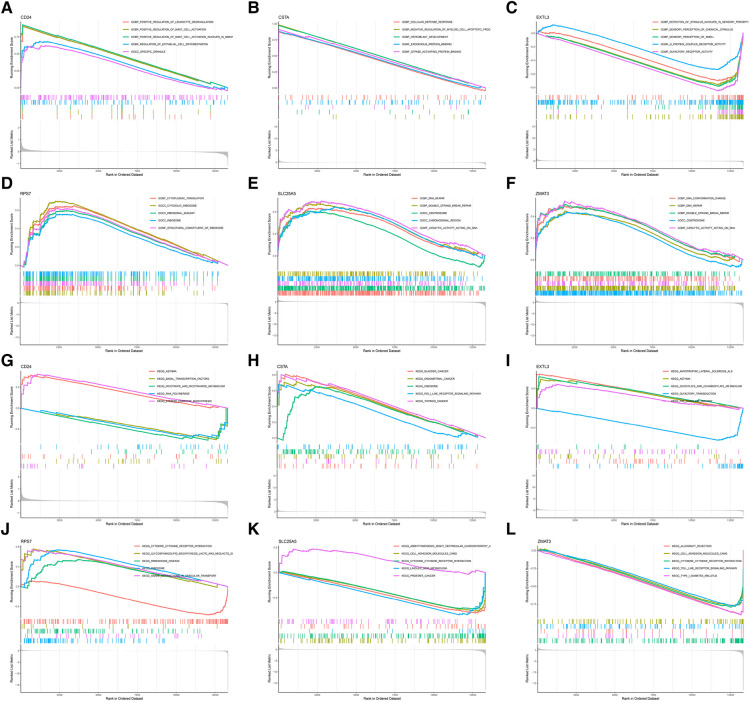
Single-gene GSEA analysis of 6 hub S-DEGs. (**A**) Single-gene GSEA GO analysis of CD24. (**B**) Single-gene GSEA GO analysis of CSTA. (**C**) Single-gene GSEA GO analysis of EXTL3. (**D**) Single-gene GSEA GO analysis of RPS7. (**E**) Single-gene GSEA GO analysis of SLC25A5. (**F**) Single-gene GSEA GO analysis of ZMAT3. (**G**) Single-gene GSEA KEGG analysis of CD24. (**H**) Single-gene GSEA KEGG analysis of CSTA. (**I**) Single-gene GSEA KEGG analysis of EXTL3. (**J**) Single-gene GSEA KEGG analysis of RPS7. (**K**) Single-gene GSEA KEGG analysis of SLC25A5. (**L**) Single-gene GSEA KEGG analysis of ZMAT3.

**Figure 7:**
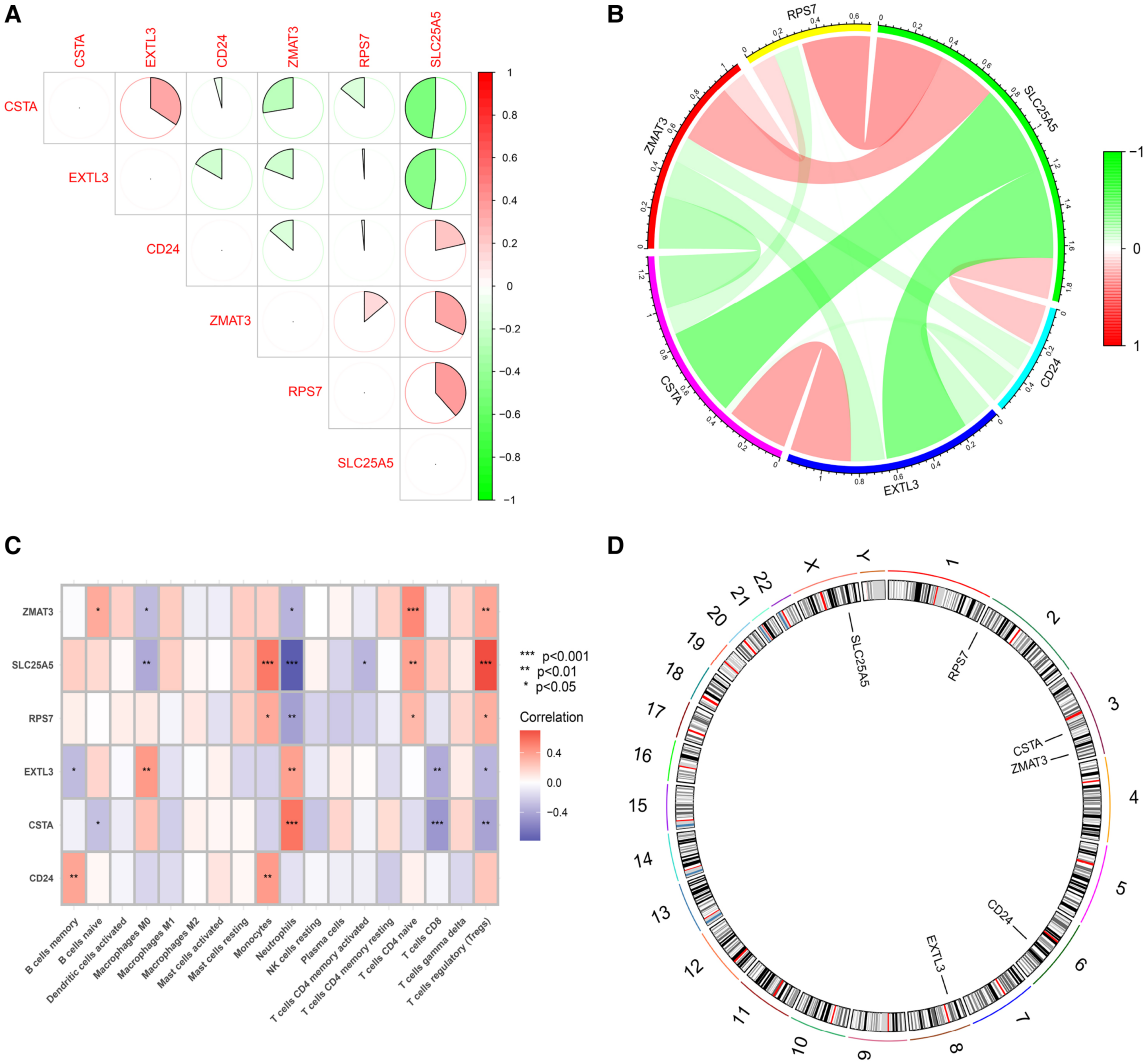
Functional analysis of 6 hub S-DEGs. (**A,B**) The correlation analysis of 6 hub S-DEGs. (**C**) The correlation analysis of infiltrated immune cells and 6 hub S-DEGs. (**D**) The position of 6 hub S-DEGs on the chromosome.

In order to further explore the main biological functions and signaling pathways affected by the 6 hub S-DEGs, the single-gene GSEA analysis was performed. The results showed that most of these hub S-DEGs are involved in regulating immune inflammatory responses. For example, CD24 gene is involved in the process of leukocyte granulation and mast cell activation, RPS7 gene, SLC25A5 gene and ZMAT3 gene are involved in the process of cytokine-cytokine receptor interaction (Figures 6A–L). In view of the close association between these hub S-DEGs and immune inflammation, we analyzed the correlation between the expression levels of these genes and the infiltration levels of immune cells. The results showed that the expression levels of these genes were correlated with each other, and with the infiltration levels of multiple immune cells (Figures 7A–C). In addition, the positions of these genes in the chromosome were shown in the Figure 7D.

### 3.5. Identification of molecular subtype for myocardial infarction based on hub S-DEGs

**Figure 8:**
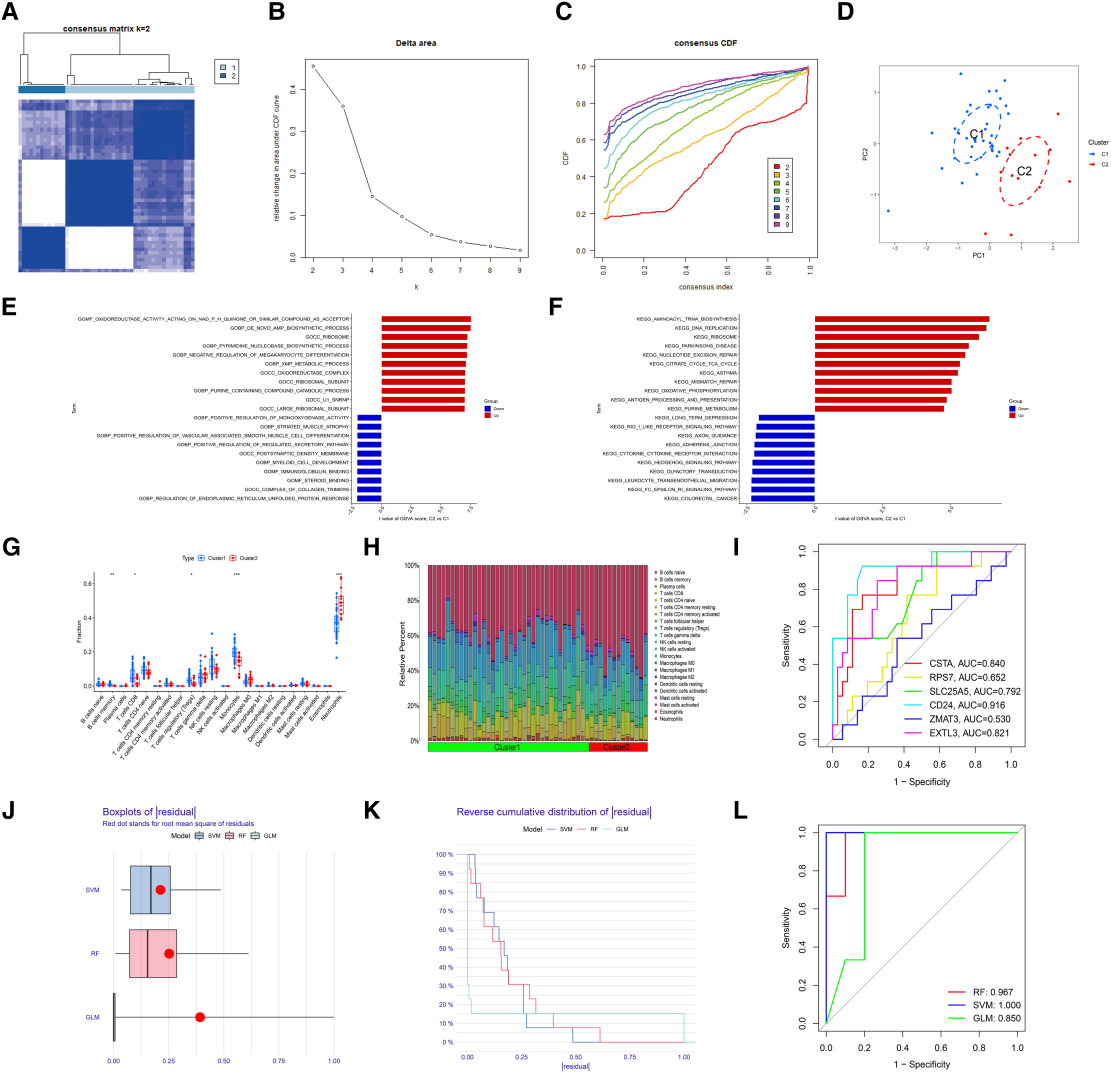
Identification of myocardial infarction molecular subtypes clusters and evaluation of the molecular subtype identification value of 6 hub S-DEGs. (**A**) The consensus clustering matrix when *k* = 2. (**B**) The CDF curves. (**C**) The CDF delta area curves. (**D**) The PCA analysis of the 2 molecular subtypes clusters. (**E**) The results of GSVA analysis showing the differences in biological functions between cluster 1 and cluster 2 (red bars represent activation of these biological functions in the cluster 2, blue bars represent inhibition of these biological functions in the cluster 2). (**F**) The results of GSVA analysis showing the differences in signaling pathways between cluster 1 and cluster 2 (red bars represent activation of these signal pathways in the cluster 2, blue bars represent inhibition of these signal pathways in the cluster 2). (**G**) The box plot showing the differences in infiltrated immune cells between cluster 1 and cluster 2. (**H**) The bar plot showing relative proportion of 22 infiltrated immune cells in cluster 1 and cluster 2. (**I**) Evaluation of the molecular subtype identification value of 6 hub S-DEGs individually by ROC analysis. (**J**) The residual boxplots of 3 machine learning models. (**K**) The cumulative residual distribution curves of 3 machine learning models. (**L**) The ROC curves of 3 machine learning models.

To identify the molecular subtype in myocardial infarction, we performed the unsupervised clustering analysis of myocardial infarction patients in the training set based on the expression levels of 6 hub S-DEGs. The results of consensus clustering matrix, CDF curves, CDF delta area curves and consensus clustering score all showed that when the *k* value is set as 2 (*k* = 2), the number of clusters is the most stable (Figures 8A–C, Supplementary Figures S3A–G and S4A–I). Therefore, we divided the myocardial infarction patients of training set into 2 molecular subtype clusters (cluster 1 and cluster 2). The PCA analysis results showed that the 2 clusters were clearly separated (Figure 8D). Since hub S-DEGs are associated with immune inflammation, we next sought to explore whether there are differences in immune inflammatory responses between the 2 molecular subtype clusters. The GSVA analysis results of the 2 clusters showed that significant differences in multiple biological processes related to immune inflammation, such as immunoglobulin binding, cytokine-cytokine receptor interaction and leukocyte transendothelial migration (Figures 8E,F). The immune cell infiltration analysis results of the 2 clusters showed that there were significant differences in the infiltration levels of memory B cell, CD8^+^ T cell, regulatory T cell, monocyte and neutrophil (Figures 8G,H). Finally, we investigated the value of these 6 hub S-DEGs in recognizing 2 molecular subtypes clusters. The single gene ROC analysis results showed that some genes had well recognition value (Figure 8I) and the machine learning model based on the 6 genes showed more satisfactory recognition value (Figures 8J–L). These results suggest that hub S-DEGs contribute to the identification of molecular subtypes associated with immune inflammation in patients with myocardial infarction. Since immune inflammation is the shared pathogenesis of myocardial infarction and depression, this molecular subtype classification method may help to identify people at high risk of myocardial infarction complicating depression.

### 3.6. Verification of the diagnostic value and molecular subtype identification value of hub S-DEGs

**Figure 9:**
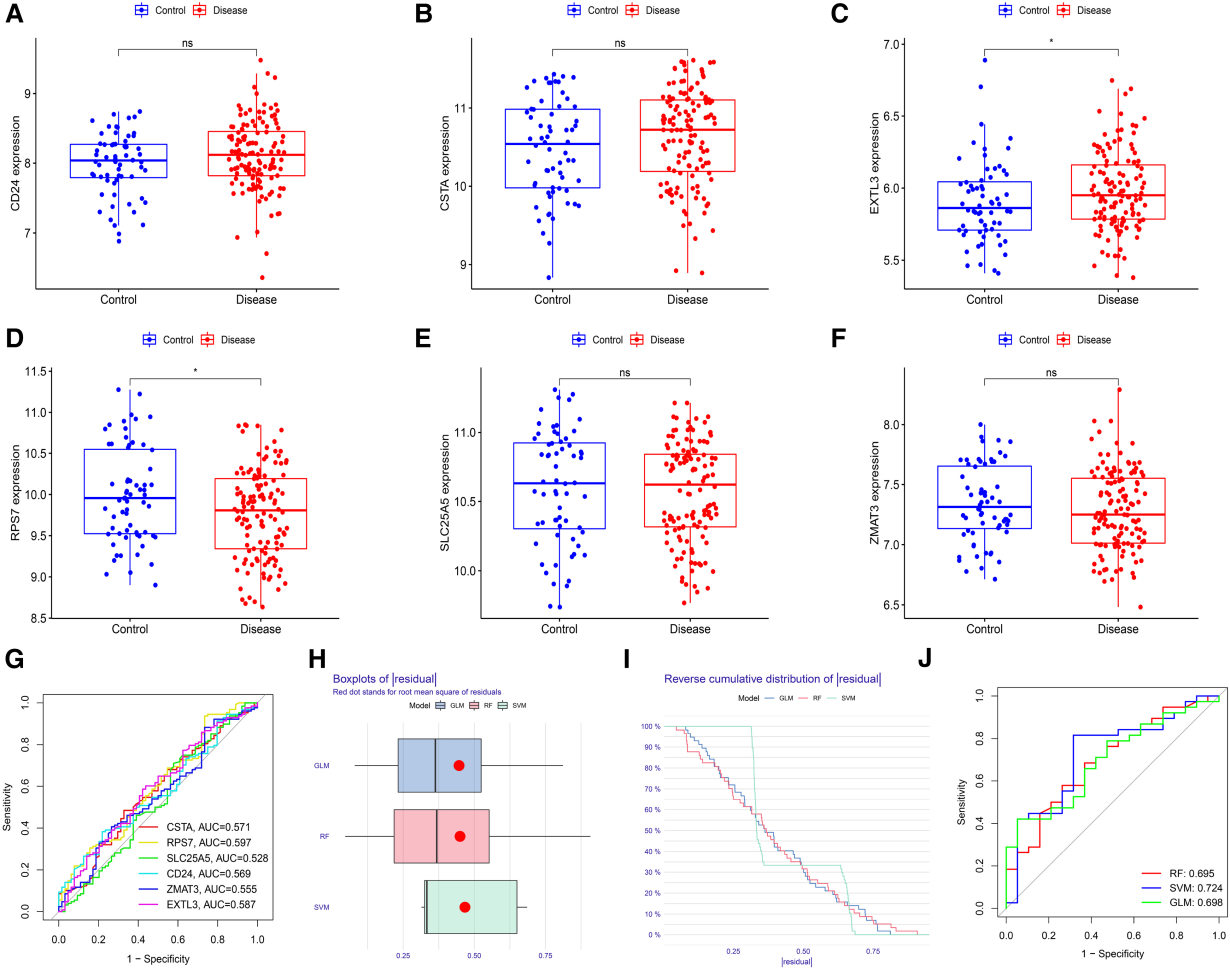
Evaluation of the expression levels and diagnostic value of 6 hub S-DEGs in the depression validation set (GSE98793). (**A–F**) Differences in expression levels of 6 hub S-DEGs between depression group and control group in the validation set. (**G**) Evaluation of the diagnostic value of 6 hub S-DEGs individually by ROC analysis in the validation set. (**H**) The residual boxplots of 3 machine learning models in the validation set. (**I**) The cumulative residual distribution curves of 3 machine learning models in the validation set. (**J**) The ROC curves of 3 machine learning models in the validation set.

**Figure 10:**
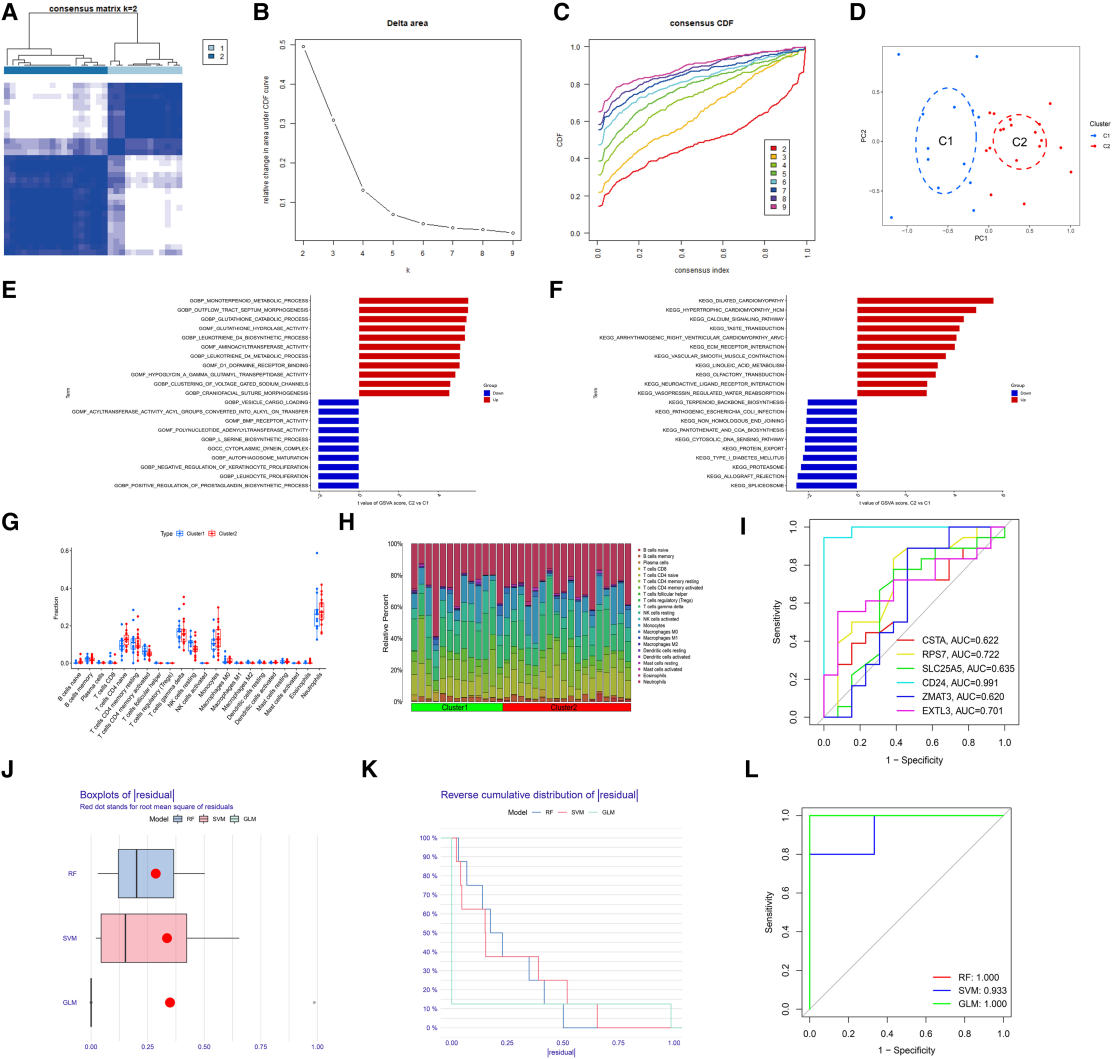
Evaluation of the molecular subtype identification value of 6 hub S-DEGs in the myocardial infarction validation set (GSE48060). (**A**) The consensus clustering matrix when *k* = 2 in the validation set. (**B**) The CDF curves in the validation set. (**C**) The CDF delta area curves in the validation set. (**D**) The PCA analysis of the 2 molecular subtypes clusters in the validation set. (**E**) The results of GSVA analysis showing the differences in biological functions between cluster 1 and cluster 2 in the validation set. (**F**) The results of GSVA analysis showing the differences in signaling pathways between cluster 1 and cluster 2 in the validation set. (**G**) The box plot showing the differences in infiltrated immune cells between cluster 1 and cluster 2 in the validation set. (**H**) The bar plot showing relative proportion of 22 infiltrated immune cells in cluster 1 and cluster 2 in the validation set. (**I**) Evaluation of the molecular subtypes identification value of 3 M-CRGs individually by ROC analysis in the validation set. (**J**) The residual boxplots of 3 machine learning models in the validation set. (**K**) The cumulative residual distribution curves of 3 machine learning models in the validation set. (**L**) The ROC curves of 3 machine learning models in the validation set.

**Figure 11:**
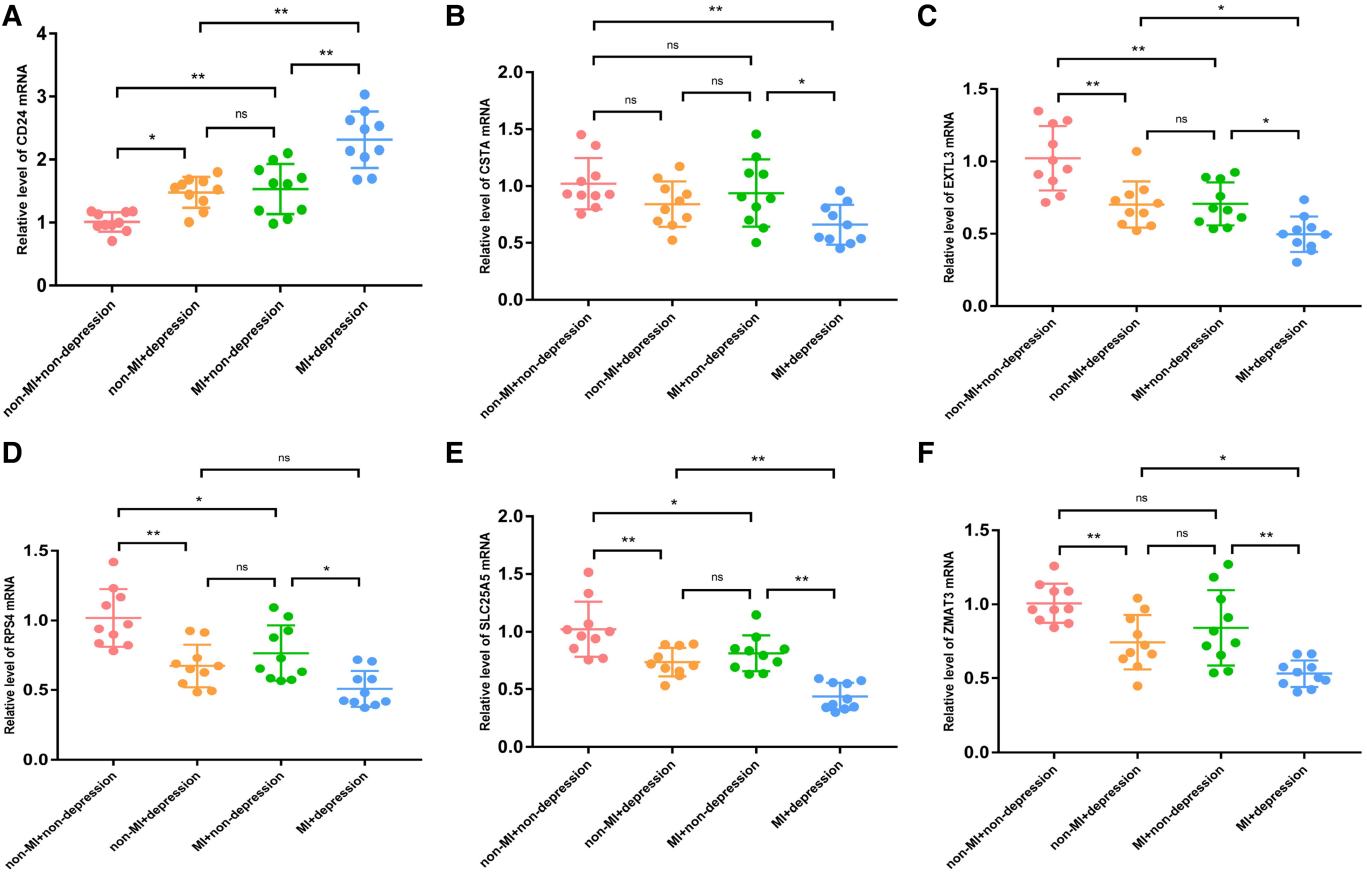
The RT-qPCR results of blood samples of 40 patients. (**A**) The mRNA expression levels of CD24. (**B**) The mRNA expression levels of CSTA. (**C**) The mRNA expression levels of EXTL3. (**D**) The mRNA expression levels of RPS4. (**E**) The mRNA expression levels of SLC25A5. (**F**) The mRNA expression levels of ZMAT3. (*P < 0.05, **P < 0.01).

First, we verified the expression levels and diagnostic value of 6 hub S-DEGs in the depression validation set (GSE98793). The results showed that the expression trends of 4 genes (CD24, RPS7, SLC25A5 and ZMAT3) in the validation set were consistent with those in the training set (Figures 9A–F). Next, we evaluated the diagnostic value of the 6 hub S-DEGs for depression in the validation set individually, and the results showed that all the individual genes had low diagnostic efficacy (Figure 9G). Subsequently, we constructed machine learning models based on these hub S-DEGs in the validation set, and the results showed that the diagnostic efficiency of SVM was significantly higher than that of the single gene, suggesting that the combined diagnostic value of these 6 hub S-DEGs for depression was also satisfactory in the validation set (Figures 9H–J). After that, we also verified the molecular subtype identification value of these genes in the myocardial infarction validation set (GSE48060). The results of consensus clustering matrix, CDF curves, CDF delta area curves and consensus clustering score all showed that when the *k* value is set as 2 (*k* = 2), the number of clusters is the most stable (Figures 10A–C, Supplementary Figures S5A–G and S6A–I). The PCA analysis results showed that the 2 clusters were clearly separated (Figure 10D). The results of GSVA showed significant differences in leukocyte proliferation between the 2 clusters (Figures 10E,F). The immune cell infiltration analysis results of the 2 clusters showed that there were certain differences in the infiltration levels of CD4^+^ T cell, natural killer cell, monocyte and neutrophil, even if the differences did not reach statistical significance (Figures 10G,H). The single gene ROC analysis results showed that some genes had well recognition value of myocardial infarction molecular subtype (Figure 10I) and the machine learning model based on the 6 genes showed more satisfactory recognition value (Figures 10J–L). Finally, we performed RT-qPCR on 40 blood samples to further verify the changes in expression levels of 6 hub S-DEGs in myocardial infarction and depression. The results found that, compared with the control group, CD24 gene showed an up-regulated trend in both myocardial infarction group and depression group, and showed further up-regulated trend in the myocardial infarction complicating depression group, while the other 5 genes showed an opposite trend (Figures 11A–F). These results were consistent with the analysis results of the training set.

### 3.7. Regulatory molecular prediction of hub S-DEGs

**Figure 12:**
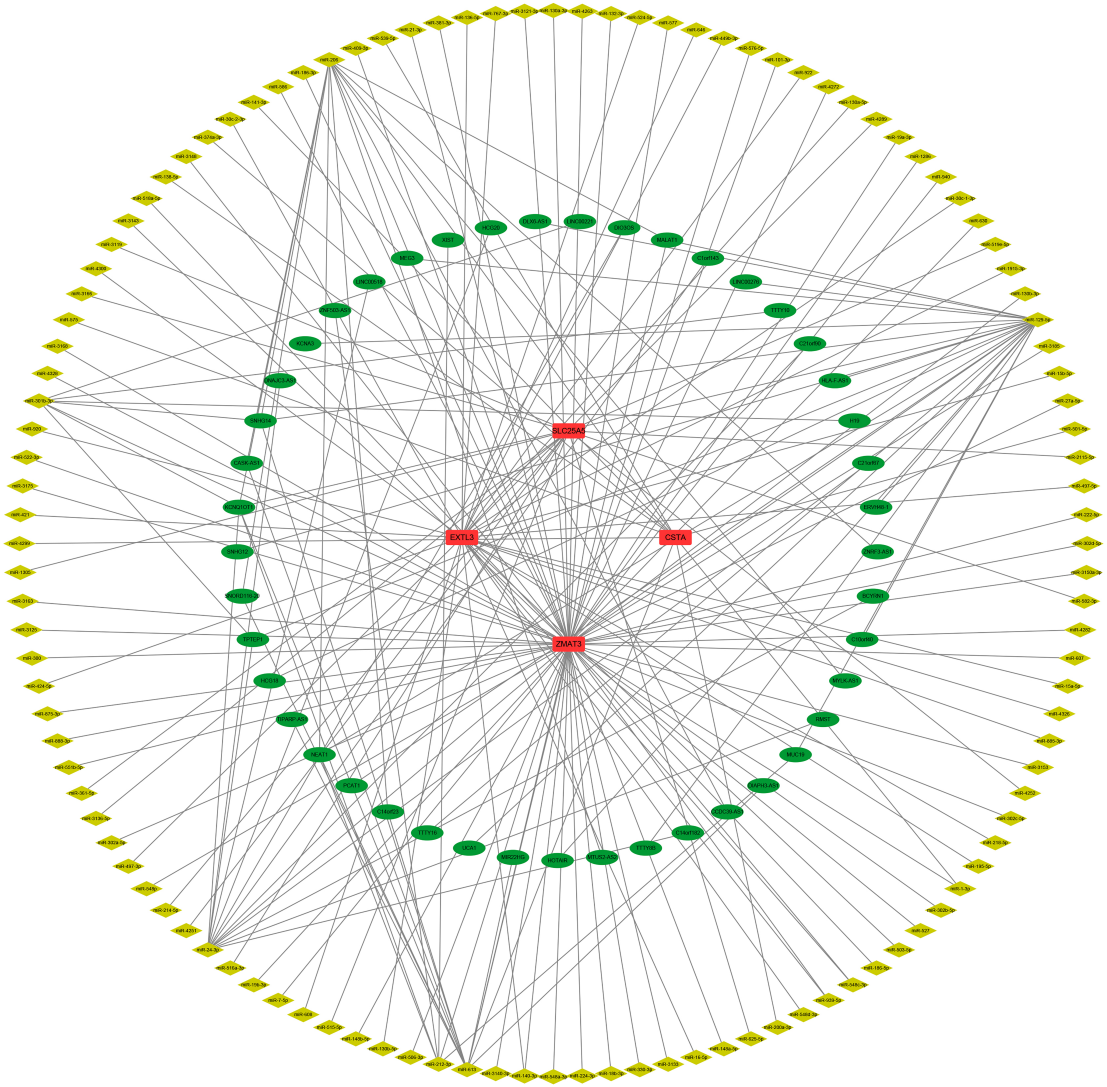
Prediction of ceRNA regulatory network of hub S-DEGs (165 nodes and 186 edges).

**Figure 13:**
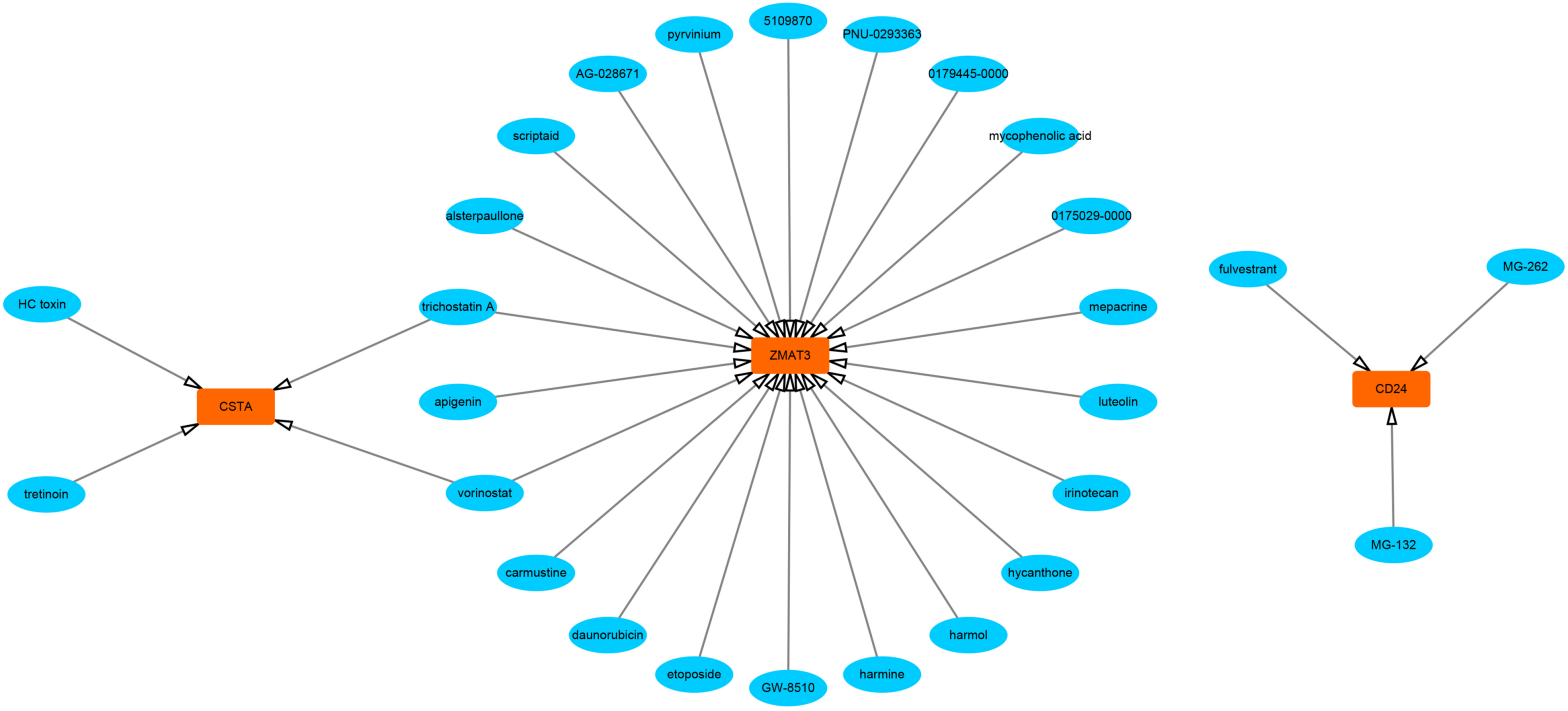
Prediction of targeted drugs of hub S-DEGs (27 drugs and 3 genes).

**Figure 14:**
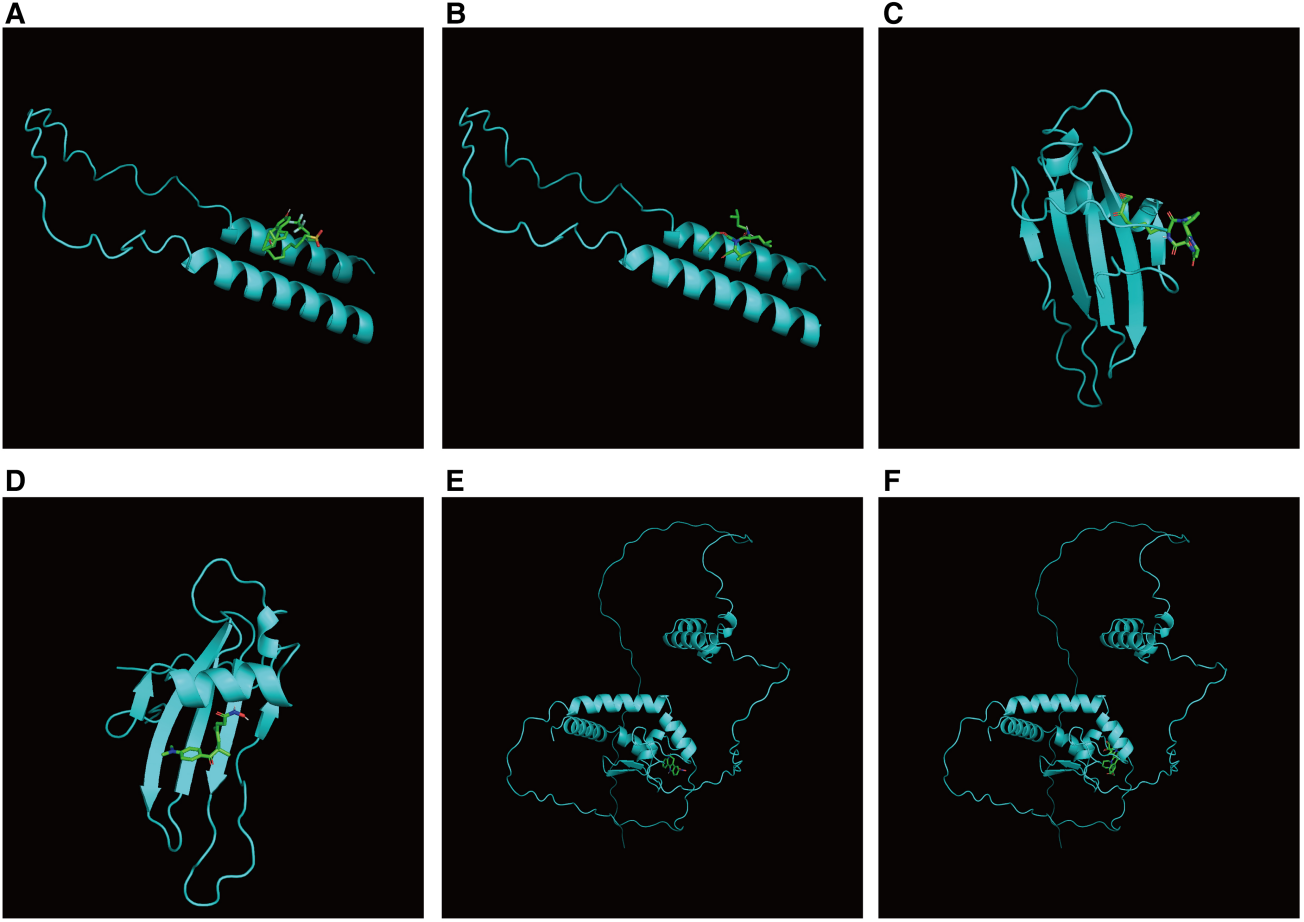
Prediction of binding sites between genes and drugs. (**A**) Prediction of binding site between CD24 and fulvestrant (binding free energy −5.5). (**B**) Prediction of binding site between CD24 and MG-132 (binding free energy −5.4). (**C**) Prediction of binding site between CSTA and HC toxin (binding free energy −7.4). (**D**) Prediction of binding site between CSTA and trichostatin A (binding free energy −6.9). (**E**) Prediction of binding site between ZMAT3 and alsterpaullone (binding free energy −7.1). (**F**) Prediction of binding site between ZMAT3 and irinotecan (binding free energy −7.4).

First, we constructed a ceRNA network based on 6 hub S-DEGs. The network included 165 nodes (4 mRNA, 116 miRNAs and 45 lncRNAs) and 186 edges (Figure 12). The interactions of each mRNA, miRNA and lncRNA in the network were summarized in Supplementary Table S4. Next, we predicted the targeted drugs of 6 hub S-DEGs from the DSigDB database, and a total of 27 drugs were obtained, among which 22 drugs may target ZMAT3 gene, 4 drugs may target CSTA gene and 3 drugs may target CD24 gene (Figure 13). However, no corresponding targeted drugs were found for EXTL3 gene, RPS4 gene and SLC25A5 gene. Finally, we performed molecular docking of drugs and genes to predict their binding sites and binding free energy, and visualized the 2 drugs that bind most stably to each gene (Figures 14A–F).

## 4. Discussion

In recent years, with the establishment of the bio-psycho-social medical model, the relationship between cardiovascular disease and mental disease has attracted more and more attention. Myocardial infarction is one of the most serious cardiovascular diseases, and its occurrence and development are closely related to psychological factors. Myocardial infarction complicating depression not only leads to decreased quality of life in patients, but also increases the incidence of adverse events and mortality (18). However, myocardial infarction complicating depression is still in a state of low recognition rate and low treatment rate. One of the main reasons for this phenomenon is that the diagnosis of depression relies on scale scores and lacks objective biomarkers (19). Once patients intentionally or unintentionally conceal their true mental state, it is difficult for doctors to make accurate judgments. In terms of treatment, there is no specific treatment for myocardial infarction complicating depression. Treatment measures for general depression, including cognitive behavioral therapy (20), exercise therapy (21), and selective serotonin reuptake inhibitors (22–24), have been shown to improve depressive mood in some studies of myocardial infarction complicating depression, but the overall efficacy is still controversial. More importantly, none of these treatments had significant effects on the long-term prognosis of myocardial infarction complicating depression patients. This may be due to the particularity of the pathogenesis of myocardial infarction complicating depression, which requires in-depth research to find more valuable targeted therapeutic measures. Therefore, it can be seen that there are still many unsolved problems in the research field of myocardial infarction complicating depression. To our knowledge, this study is the first to use bioinformatics analysis techniques to integrate and analyze multiple human blood samples with myocardial infarction or depression in an attempt to find solutions to the current challenges.

In this study, a comprehensive analysis of 4 GEO datasets, including differential analysis, GO analysis, KEGG analysis, GSVA analysis, GSEA analysis, immune cell infiltration analysis, and correlation analysis, found that immune inflammatory response may be the shared pathogenesis of myocardial infarction and depression, which is consistent with the results of previous studies. Many results of previous studies have supported immune inflammation as the central link between myocardial infarction and depression. On the one hand, both the onset of myocardial infarction and depression are associated with the abnormal activation of the immune inflammatory system, and the levels of immune cells or inflammatory factors in the blood and tissues of patients are significantly up-regulated (25, 26). On the other hand, the immune inflammatory response caused by myocardial infarction and depression can influence each other to cause the occurrence or exacerbation of the diseases. For example, the activated immune inflammatory response after myocardial infarction can change the permeability of the blood-brain barrier and induce neuroinflammatory response in the brain, which may lead to depression (27). Depression, in turn, can aggravate the immune inflammatory response of myocardial cells and vascular endothelial cells, and accelerate the death process of myocardial cells after myocardial infarction (28). However, previous studies on the pathogenesis of myocardial infarction complicating depression were all conducted by basic experimental methods. This study further enriched the pathogenesis of myocardial infarction complicating depression from the clinical level by bioinformatics analysis of transcriptomics of patients. In addition, this study identified 6 hub S-DEGs that were significantly differentially expressed in both the myocardial infarction and depression training sets. The machine learning results showed that these genes are not only valuable in the diagnosis of depression, but also have potential in the identification of molecular subtype of myocardial infarction. As we all know, the diagnosis of myocardial infarction has objective blood biomarkers, while depression does not. After myocardial infarction, how to early identify patients with depression or high-risk patients prone to depression is crucial. The results of this study found that these hub genes contribute to the identification of myocardial infarction patients who have developed depression. Previous studies have found that disease can be divided into different molecular subtypes, and these molecular subtypes have significant differences in clinical manifestations, treatment sensitivity and prognosis (29, 30). In this study, we found that 6 hub S-DEGs were able to identify two distinct clusters of molecular subtypes in patients with myocardial infarction that differ in a variety of immunoinflammatory related biological functions. This suggests that the hub S-DEGs are promising for identifying subgroups population with specific immunoinflammatory characteristics in patients with myocardial infarction. Since immune inflammation is the common pathogenesis of myocardial infarction and depression, different subgroups population may have different risks of depression. Therefore, for those patients with myocardial infarction who have not yet developed significant depressive symptoms, the molecular subtype identification value of these hub S-DEGs may help to identify people at high risk of depression. In addition, since these 6 hub S-DEGs are the differential genes shared by myocardial infarction and depression, they may be key therapeutic targets for myocardial infarction complicating depression. Therefore, the ceRNA regulatory network and targeted drug regulatory network of these genes were constructed based on the public database, and the binding site and binding strength of drugs were predicted by molecular docking technology, in order to provide a valuable reference for the targeted therapy of myocardial infarction complicating depression. It should be noted that although the diagnostic value and molecular subtype identification value of these genes were verified in the validation set, the expression trends of a few of them (CSTA and EXTL3) were not completely consistent in the training set and validation set. By comparing the differences between these datasets, we found that some of the depressed patients in the validation set also had anxiety state, which may be the main reason for this result. Therefore, in order to further verify the changes in the expression levels of these genes in myocardial infarction and depression, we collected blood samples of 40 patients and performed RT-qPCR experiments. The results showed that the changes in the expression levels of these genes were basically consistent with the trends of training set. In addition, it is important to note that, when performing bioinformatics analysis, the differential analysis between tumor tissue and tumor adjacent tissue usually results in many differential genes. However, in non-tumor diseases, especially when the blood samples of non-tumor diseases were used for differential analysis, fewer differential genes are usually obtained. In this case, it is sometimes not appropriate to use stringent screening criteria, such as adjusted *P*-value less than 0.05 and |log2 fold change| >0.585. Some previous bioinformatics studies used *P*-value less than 0.05 or |log2 fold change| >0.2 as the screening condition for differential genes (31–33). Therefore, in our study, the *P*-value less than 0.05 and |log2 fold change| >0.2 was used as a differential gene screening condition. However, it should be admitted that this screening condition would lead to a higher false positive rate. Another point to be noted is that the weighted gene co-expression network analysis (WGCNA) is a commonly used analytical method to identify differential genes for certain diseases. When we conducted this study, we also tried to use this analytical method. However, we found that no significantly differential gene modules could be identified using WGCNA in training sets or validation sets (Supplementary Figures S7A–F). Based on the previously research, other differential analysis methods could be used instead of WGCNA in this case (34, 35). Admittedly, this may affect the number of differential genes obtained in this study. We look forward to more high-quality sequencing or microarray data related to myocardial infarction or depression in the future to make up for the unfinished work of our study.

Cluster of differentiation 24 (CD24) is a glycoprotein that attaches to the cell surface, and its way of anchoring cells is mediated by glycosylphosphatidylinositol (36). CD24 plays an important role in regulating immune inflammatory responses associated with B and T cells (37). It was found that the level of CD24hi cells was correlated with the occurrence of myocardial infarction (38). Meanwhile, animal experiments showed that CD24 gene knockout improved anxiety-like behavior and cognitive performance in mice (39). In our study, the expression level of CD24 was also found to be increased in blood samples of patients with depression, suggesting that CD24 may be potentially related to emotional regulation. Cystatin A (CSTA), a cysteine protease inhibitor, is a key precursor protein that constitutes the cornified cell envelope of keratinocytes and has been proven to play an important role in epidermal development as well as invasion and metastasis of various cancers (40). Interestingly, a previous study had shown a negative correlation between the level of CSTA in tissues and depressive symptoms (41). This result is consistent with the detection results of blood samples in the depression training set in our study, further suggesting that CSTA is related to depression and may be a potential biomarker for depression diagnosis. Exostosin like glycosyltransferase 3 (EXTL3) gene encodes a glycosyltransferase protein, which is closely related to the synthesis of heparin and heparin sulfate, and plays an important role in maintaining the basic functions of organisms (42). A recent study found that reduced levels of a specific glycosyltransferase enzyme in the brain caused depression-like emotions in mice (43). Our results also showed that in both the training set and validation set for depression, the expression levels of EXTL3 in the depression group were lower than those in the control group, further confirming the association between glycosyltransferase and depression. However, we found no studies that reported the role of glycosyltransferase in myocardial infarction. Further research on the function of glycosyltransferase is expected to reveal the common pathogenesis of myocardial infarction and depression. Ribosomal protein S7 (RPS7) is an important component of the 40S subunit of the ribosome and is closely related to protein synthesis (44). Although few studies have reported the relationship between RPS7 and myocardial infarction or depression, other members of the ribosomal protein family, such as P70S6K, have been reported to be significantly down-regulated in both myocardial infarction and depression mice (45, 46). These results are similar to the results of our study, suggesting that ribosomal protein may be involved in the pathogenesis of both myocardial infarction and depression, and is a potential biomarker of myocardial infarction complicating depression. However, it is worth noting that the expression of ribosomal proteins RPL17 and RPL34 in the blood samples of patients with depression has been reported to be up-regulated (47), indicating that the changes in the expression level of each member of the ribosomal protein family in the disease are not completely consistent, and the changes in the expression level of RPS7 in myocardial infarction and depression need further confirmation. The main function of SLC25A5 is to regulate the transfer of ADP and ATP in cytoplasm and mitochondrial matrix, which acts as a kind of gating (48). It was found that SLC25A5 was significantly down-regulated in the midbrain raphe nuclei of mice subjected to prolonged stress (49). Our results also showed that the expression level of SLC25A5 in the blood of depression patients was low, suggesting that SLC25A5 may be related to negative mood. Zinc finger matrin-type 3 (ZMAT3) gene encodes an RNA-binding protein containing zinc finger domains and nuclear localization signals. ZMAT3 could affect the stability and translation function of RNA by regulating the alternative splicing process, which plays an important role in the post-transcriptional regulation of genes (50). Most of the previous studies on ZMAT3 have focused on the oncology domain (51). The results of a recent study indicate that ZMAT2, a gene from the same family as ZMAT3, is a significant transcriptome-wide risk gene for depression and shows a strong association with depression in the brain expression quantitative loci data set (52). This finding, together with our results, suggests that zinc finger matrin family genes are related to depression to some extent, and the specific mechanism needs to be further studied in the future.

This investigation integrated 4 studies with large sample sizes in hematologic transcriptomics of myocardial infarction and depression to date. In addition, blood samples from myocardial infarction complicating depression patients were collected to further validate the analysis results of the public database. Our study not only provided relatively reliable evidence for the development of diagnostic markers and targeted therapeutic measures for myocardial infarction complicating depression, but also explored a new direction for future research on the pathogenesis. However, it should be acknowledged that this study has the following limitations: First, the pathogenesis of myocardial infarction complicating depression has been studied through the enrichment analysis of overlapping genes, and the therapeutic drugs and non-coding RNA targeting hub genes have been predicted by public databases. However, there is a lack of *in vitro* and *in vivo* experiments to verify these results. Second, the small number of clinical samples collected in RT-qPCR validation experiments may affect the rigor of the results. We believe that these limitations will be addressed in our further studies in the future.

Overall, through bioinformatics analysis and clinical sample validation, this study explored the potential pathogenesis of myocardial infarction complicating depression, found the hub genes with diagnostic and molecular subtype identification value, and predicted the potential non-coding RNA and therapeutic drugs that could target and regulate these hub genes. This study has explored a new research direction for the field of myocardial infarction complicating depression, further research on this basis is expected to make more exciting breakthroughs in the pathogenesis interpretation, early diagnosis and individualized treatment of myocardial infarction complicating depression in the future.

## Data Availability

The original contributions presented in the study are included in the article/**Supplementary Material**, further inquiries can be directed to the corresponding authors.
