# Supplementary material for: Investigation of the shared molecular mechanisms and hub genes between myocardial infarction and depression
Source: Front Cardiovasc Med. 2023 Jul 21;10:1203168. doi: 10.3389/fcvm.2023.1203168 (PMC10401437; doi:10.3389/fcvm.2023.1203168)
Supplement: Supplementary file 1 [file Table1.docx]

**Supplementary Table S1. Summary of four GEO datasets**

| **Name** | **Disease Type** | **Disease Samples** | **Control Samples** | **Group** |
| --- | --- | --- | --- | --- |
| GSE34198 | Myocardial infarction | 49 | 48 | Training set |
| GSE19738 | Depression | 33 | 34 | Training set |
| GSE48060 | Myocardial infarction | 31 | 21 | Validation set |
| GSE98793 | Depression | 128 | 64 | Validation set |

**Supplementary Table S2. Baseline characteristics**

|  | **Control** | **MI** | **Depression** | **MI complicating depression** | ***P*** |
| --- | --- | --- | --- | --- | --- |
| Age | 66.00±8.58 | 67.70±5.78 | 68.60±4.62 | 71.60±7.21 | 0.3163 |
| Gender  (Male / Female) | 5 / 5 | 7 / 3 | 3 / 7 | 4 / 6 | 0.3196 |
| BMI  (kg/m^2^) | 24.76±2.63 | 25.14±2.81 | 25.28±3.00 | 25.53±3.04 | 0.9437 |
| Smoking  (Yes / No) | 3 / 7 | 7 / 3 | 4 / 6 | 4 / 6 | 0.3035 |
| Hypertension  (Yes / No) | 5 / 5 | 8 / 2 | 6 / 4 | 8 / 2 | 0.3799 |
| Diabetes  (Yes / No) | 2 / 8 | 8 / 2 | 3 / 7 | 9 / 1 | 0.0019 |
| Stroke  (Yes / No) | 2 / 8 | 5 / 5 | 4 / 6 | 6 / 4 | 0.3105 |
| Heart failure  (Yes / No) | 2 / 8 | 9 / 1 | 3 / 7 | 10 / 0 | 0.0001 |
|  | Note: BMI: body mass index. | | | | |

**Supplementary Table S3. Primer sequences**

| **Name** | **Species** | **Forward primer** | **Reverse primer** |
| --- | --- | --- | --- |
| CD24 | Human | CTCCTACCCACGCAGATTTATTC | AGAGTGAGACCACGAAGAGAC |
| CSTA | Human | AAACCCGCCACTCCAGAAATC | CACCTGCTCGTACCTTAATGTAG |
| EXTL3 | Human | CTCTGGCTTCCCGGTCTAC | AGGCGATGTCTGCATTTTCTG |
| RPS7 | Human | CCAAGTCCGGCTAGTACGC | TCGAGTTGGCTTAGGCAGAAT |
| SLC25A5 | Human | TTATAGACTGCGTGGTCCGTA | GGCGAAGTTAAGAGCCTGGG |
| ZMAT3 | Human | CCTTACTTCAATCCCCGCTCT | CTTCGCCAGCTCCAACATTAC |
| GAPDH | Human | GGACCTGACCTGCCGTCTAG | GTAGCCCAGGATGCCCTTGA |

**Supplementary Table S4. Interactions of ceRNA**

| **Name of miRNA** | **Name of interacting**  **miRNA-mRNA or lncRNA-miRNA** | **Interaction type** |
| --- | --- | --- |
| miR-3150a-3p  miR-3185  miR-1286  miR-101-3p  miR-4263  miR-409-3p  miR-575  miR-1305  miR-551b-5p  miR-4251  miR-148b-5p  miR-548a-3p  miR-625-5p  miR-527  miR-3153  miR-302d-5p  miR-129-5p  miR-19a-3p  miR-576-5p  miR-130a-3p  miR-206  miR-138-5p  miR-4328  miR-424-5p  miR-497-3p  miR-7-5p  miR-613  miR-3133  miR-939-5p  miR-195-5p  miR-15a-5p  miR-2115-5p  miR-519e-5p  miR-939-5p  miR-449b-3p  miR-3121-3p  miR-186-3p  miR-518a-5p  miR-301b-3p  miR-3163  miR-361-5p  miR-24-3p  miR-130b-5p  miR-224-3p  miR-206  miR-548c-3p  miR-218-5p  miR-607  miR-501-5p  miR-630  miR-4272  miR-548c-3p  miR-767-3p  miR-586  miR-3143  miR-920  miR-3125  miR-3136-5p  miR-516a-3p  miR-506-3p  miR-18b-3p  miR-200a-3p  miR-302b-5p  miR-885-3p  miR-222-5p  miR-130b-3p  miR-4289  miR-646  miR-136-5p  miR-141-3p  miR-3119  miR-613  miR-421  miR-875-3p  miR-548p  miR-608  miR-3140-3p  miR-16-5p  miR-186-5p  miR-302c-5p  miR-4282  miR-27a-5p  miR-30c-1-3p  miR-922  miR-132-3p  miR-539-5p  miR-3148  miR-3168  miR-4299  miR-888-3p  miR-214-5p  miR-515-5p  miR-140-3p  miR-148a-5p  miR-503-5p  miR-4252  miR-582-3p  miR-15b-5p  miR-940  miR-140-3p  miR-577  miR-381-3p  miR-30c-2-3p  miR-4300  miR-522-3p  miR-300  miR-302a-5p  miR-19b-3p  miR-212-3p  miR-330-3p  miR-548d-3p  miR-1-3p  miR-4326  miR-497-5p  miR-1915-3p  miR-130a-5p  miR-524-5p  miR-21-3p  miR-374a-3p  miR-3166  miR-3175  miR-1-3p | ZMAT3  ZMAT3  ZMAT3  ZMAT3  ZMAT3  ZMAT3  CSTA  SLC25A5  ZMAT3  SLC25A5  ZMAT3  ZMAT3  SLC25A5  ZMAT3  ZMAT3  ZMAT3  ZMAT3  ZMAT3  ZMAT3  ZMAT3  CSTA  CSTA  ZMAT3  EXTL3  EXTL3  ZMAT3  ZMAT3  ZMAT3  EXTL3  EXTL3  EXTL3  SLC25A5  SLC25A5  ZMAT3  EXTL3  ZMAT3  ZMAT3  ZMAT3  ZMAT3  ZMAT3  SLC25A5  ZMAT3  EXTL3  ZMAT3  ZMAT3  ZMAT3  EXTL3  ZMAT3  CSTA  ZMAT3  ZMAT3  EXTL3  EXTL3  ZMAT3  ZMAT3  EXTL3  ZMAT3  SLC25A5  SLC25A5  ZMAT3  ZMAT3  CSTA  ZMAT3  EXTL3  ZMAT3  ZMAT3  EXTL3  EXTL3  EXTL3  CSTA  SLC25A5  CSTA  EXTL3  ZMAT3  ZMAT3  EXTL3  ZMAT3  EXTL3  ZMAT3  ZMAT3  ZMAT3  ZMAT3  EXTL3  EXTL3  ZMAT3  CSTA  ZMAT3  ZMAT3  CSTA  ZMAT3  SLC25A5  SLC25A5  EXTL3  ZMAT3  EXTL3  SLC25A5  SLC25A5  EXTL3  ZMAT3  ZMAT3  ZMAT3  ZMAT3  EXTL3  EXTL3  ZMAT3  ZMAT3  ZMAT3  ZMAT3  ZMAT3  ZMAT3  EXTL3  ZMAT3  EXTL3  EXTL3  EXTL3  EXTL3  EXTL3  ZMAT3  CSTA  SLC25A5  ZMAT3  CSTA | miRNA-mRNA  miRNA-mRNA  miRNA-mRNA  miRNA-mRNA  miRNA-mRNA  miRNA-mRNA  miRNA-mRNA  miRNA-mRNA  miRNA-mRNA  miRNA-mRNA  miRNA-mRNA  miRNA-mRNA  miRNA-mRNA  miRNA-mRNA  miRNA-mRNA  miRNA-mRNA  miRNA-mRNA  miRNA-mRNA  miRNA-mRNA  miRNA-mRNA  miRNA-mRNA  miRNA-mRNA  miRNA-mRNA  miRNA-mRNA  miRNA-mRNA  miRNA-mRNA  miRNA-mRNA  miRNA-mRNA  miRNA-mRNA  miRNA-mRNA  miRNA-mRNA  miRNA-mRNA  miRNA-mRNA  miRNA-mRNA  miRNA-mRNA  miRNA-mRNA  miRNA-mRNA  miRNA-mRNA  miRNA-mRNA  miRNA-mRNA  miRNA-mRNA  miRNA-mRNA  miRNA-mRNA  miRNA-mRNA  miRNA-mRNA  miRNA-mRNA  miRNA-mRNA  miRNA-mRNA  miRNA-mRNA  miRNA-mRNA  miRNA-mRNA  miRNA-mRNA  miRNA-mRNA  miRNA-mRNA  miRNA-mRNA  miRNA-mRNA  miRNA-mRNA  miRNA-mRNA  miRNA-mRNA  miRNA-mRNA  miRNA-mRNA  miRNA-mRNA  miRNA-mRNA  miRNA-mRNA  miRNA-mRNA  miRNA-mRNA  miRNA-mRNA  miRNA-mRNA  miRNA-mRNA  miRNA-mRNA  miRNA-mRNA  miRNA-mRNA  miRNA-mRNA  miRNA-mRNA  miRNA-mRNA  miRNA-mRNA  miRNA-mRNA  miRNA-mRNA  miRNA-mRNA  miRNA-mRNA  miRNA-mRNA  miRNA-mRNA  miRNA-mRNA  miRNA-mRNA  miRNA-mRNA  miRNA-mRNA  miRNA-mRNA  miRNA-mRNA  miRNA-mRNA  miRNA-mRNA  miRNA-mRNA  miRNA-mRNA  miRNA-mRNA  miRNA-mRNA  miRNA-mRNA  miRNA-mRNA  miRNA-mRNA  miRNA-mRNA  miRNA-mRNA  miRNA-mRNA  miRNA-mRNA  miRNA-mRNA  miRNA-mRNA  miRNA-mRNA  miRNA-mRNA  miRNA-mRNA  miRNA-mRNA  miRNA-mRNA  miRNA-mRNA  miRNA-mRNA  miRNA-mRNA  miRNA-mRNA  miRNA-mRNA  miRNA-mRNA  miRNA-mRNA  miRNA-mRNA  miRNA-mRNA  miRNA-mRNA  miRNA-mRNA  miRNA-mRNA  miRNA-mRNA  miRNA-mRNA |
| miR-129-5p  miR-129-5p  miR-129-5p  miR-129-5p  miR-129-5p  miR-129-5p  miR-129-5p  miR-129-5p  miR-129-5p  miR-129-5p  miR-129-5p  miR-129-5p  miR-129-5p  miR-129-5p  miR-129-5p  miR-129-5p  miR-129-5p  miR-129-5p  miR-129-5p  miR-129-5p  miR-24-3p  miR-24-3p  miR-24-3p  miR-24-3p  miR-24-3p  miR-24-3p  miR-24-3p  miR-24-3p  miR-24-3p  miR-24-3p  miR-24-3p  miR-24-3p  miR-206  miR-206  miR-206  miR-206  miR-206  miR-206  miR-206  miR-206  miR-206  miR-206  miR-206  miR-301b-3p  miR-301b-3p  miR-301b-3p  miR-301b-3p  miR-301b-3p  miR-301b-3p  miR-212-3p  miR-212-3p  miR-212-3p  miR-212-3p  miR-212-3p  miR-613  miR-613  miR-613  miR-613  miR-613  miR-613  miR-613  miR-613  miR-613  miR-613 | KCNQ1OT1  TPTEP1  NEAT1  TTTY16  HOTAIR  TTTY8B  MUC19  C10orf40  ERVH48-1  HLA-F-AS1  LINC00276  MALAT1  DLX6-AS1  MEG3  KCNA3  SNHG14  SNHG12  HCG18  PCAT1  UCA1  KCNQ1OT1  TPTEP1  C14orf182  RMST  BCYRN1  C21orf67  C21orf90  C1orf143  DIO3OS  HCG20  LINC00518  DNAJC3-AS1  CASK-AS1  SNORD116-20  KCNQ1OT1  MALAT1  C14orf23  SNHG14  NEAT1  MTUS2-AS2  CCDC39-AS1  MYLK-AS1  ZNRF3-AS1  H19  SNHG14  TTTY10  TPTEP1  KCNQ1OT1  LINC00221  XIST  ZNF503-AS1  KCNQ1OT1  MUC19  NEAT1  SNORD116-20  CASK-AS1  KCNQ1OT1  TIPARP-AS1  C14orf23  MIR22HG  SNHG14  MALAT1  NEAT1  DIAPH3-AS1 | lncRNA-miRNA  lncRNA-miRNA  lncRNA-miRNA  lncRNA-miRNA  lncRNA-miRNA  lncRNA-miRNA  lncRNA-miRNA  lncRNA-miRNA  lncRNA-miRNA  lncRNA-miRNA  lncRNA-miRNA  lncRNA-miRNA  lncRNA-miRNA  lncRNA-miRNA  lncRNA-miRNA  lncRNA-miRNA  lncRNA-miRNA  lncRNA-miRNA  lncRNA-miRNA  lncRNA-miRNA  lncRNA-miRNA  lncRNA-miRNA  lncRNA-miRNA  lncRNA-miRNA  lncRNA-miRNA  lncRNA-miRNA  lncRNA-miRNA  lncRNA-miRNA  lncRNA-miRNA  lncRNA-miRNA  lncRNA-miRNA  lncRNA-miRNA  lncRNA-miRNA  lncRNA-miRNA  lncRNA-miRNA  lncRNA-miRNA  lncRNA-miRNA  lncRNA-miRNA  lncRNA-miRNA  lncRNA-miRNA  lncRNA-miRNA  lncRNA-miRNA  lncRNA-miRNA  lncRNA-miRNA  lncRNA-miRNA  lncRNA-miRNA  lncRNA-miRNA  lncRNA-miRNA  lncRNA-miRNA  lncRNA-miRNA  lncRNA-miRNA  lncRNA-miRNA  lncRNA-miRNA  lncRNA-miRNA  lncRNA-miRNA  lncRNA-miRNA  lncRNA-miRNA  lncRNA-miRNA  lncRNA-miRNA  lncRNA-miRNA  lncRNA-miRNA  lncRNA-miRNA  lncRNA-miRNA  lncRNA-miRNA |


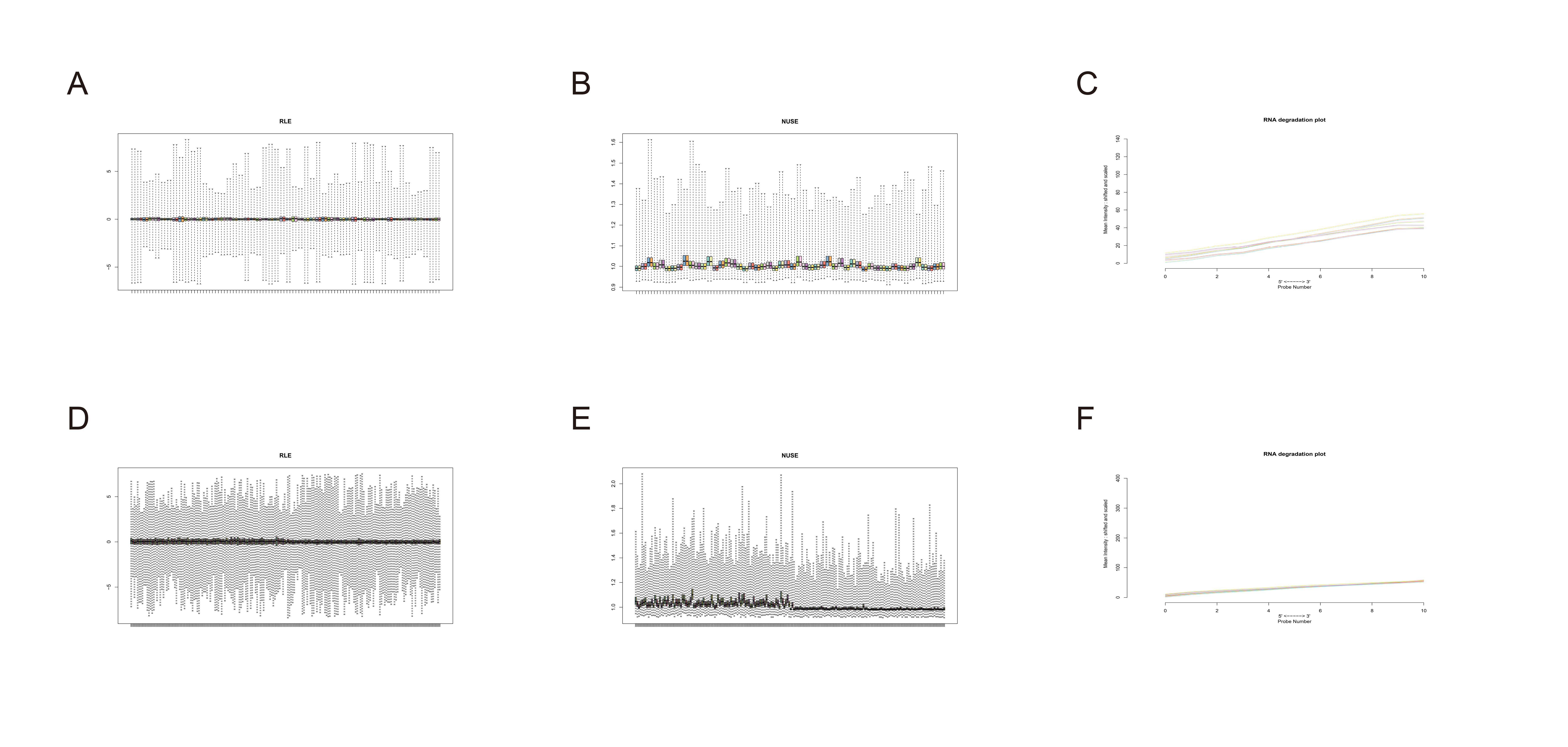


**Supplementary Figure S1.** The quality control of GSE48060 dataset and GSE98793 dataset. (A) The RLE boxplot of GSE48060 dataset. (B) The NUSE boxplot of GSE48060 dataset. (C) The RNA degradation curve of GSE48060 dataset. (D) The RLE boxplot of GSE98793 dataset. (E) The NUSE boxplot of GSE98793 dataset. (F) The RNA degradation curve of GSE98793 dataset.


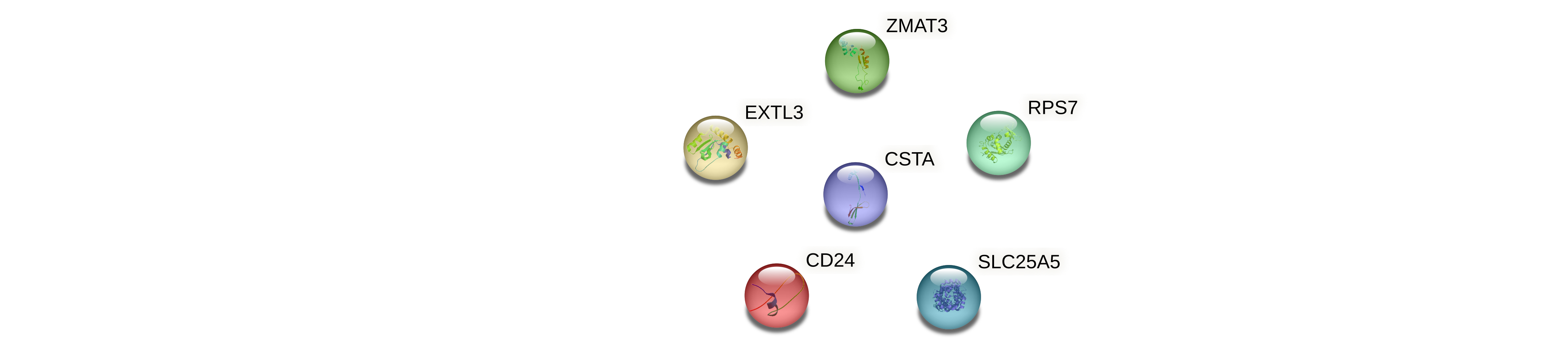


**Supplementary Figure S2.** The PPI network analysis result of 6 hub S-DEGs.


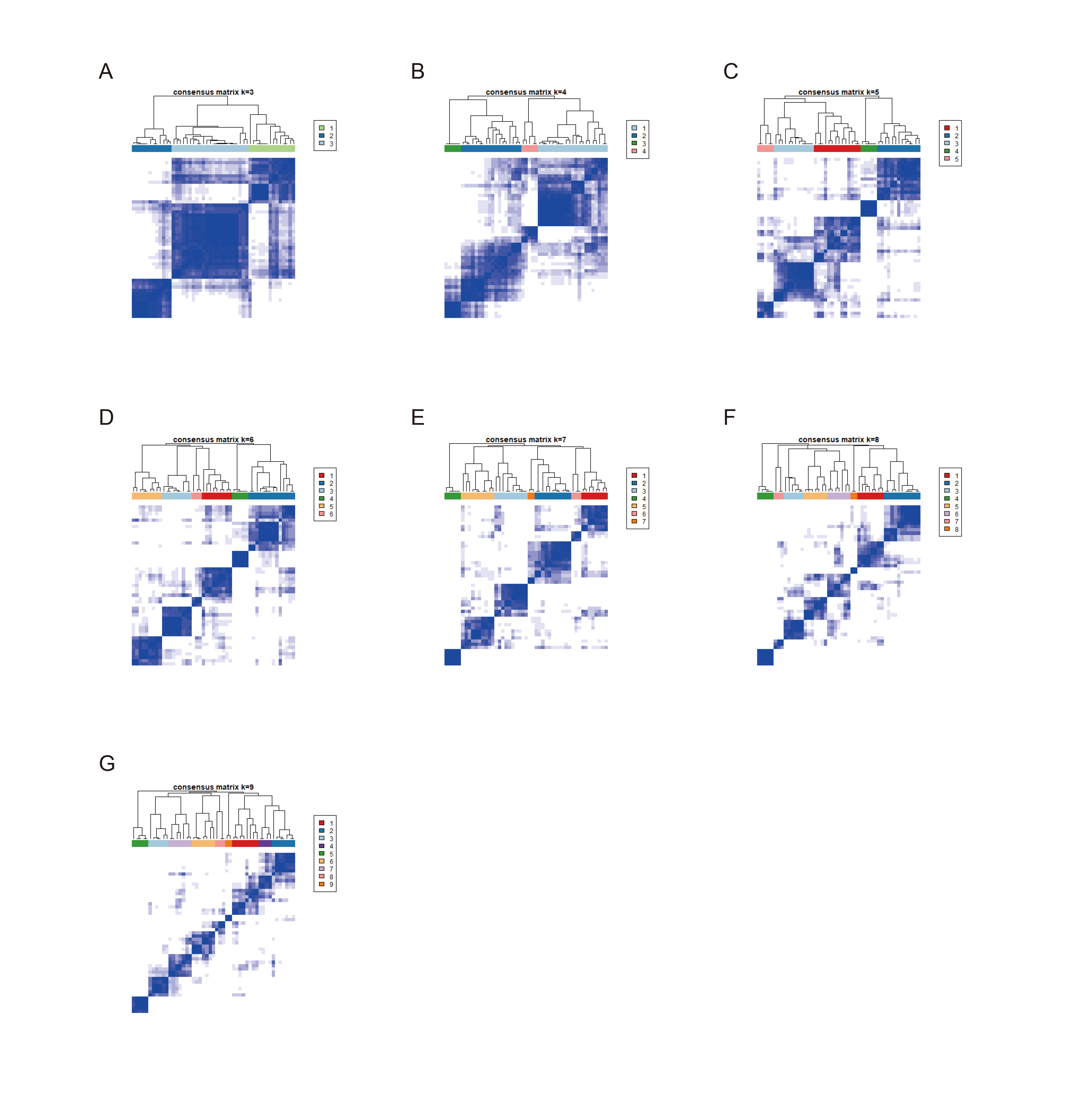


**Supplementary Figure S3.** The consensus clustering matrix when k = 3-9. (A) k = 3. (B) k = 4. (C) k = 5. (D) k = 6. (E) k = 7. (F) k = 8. (G) k = 9.


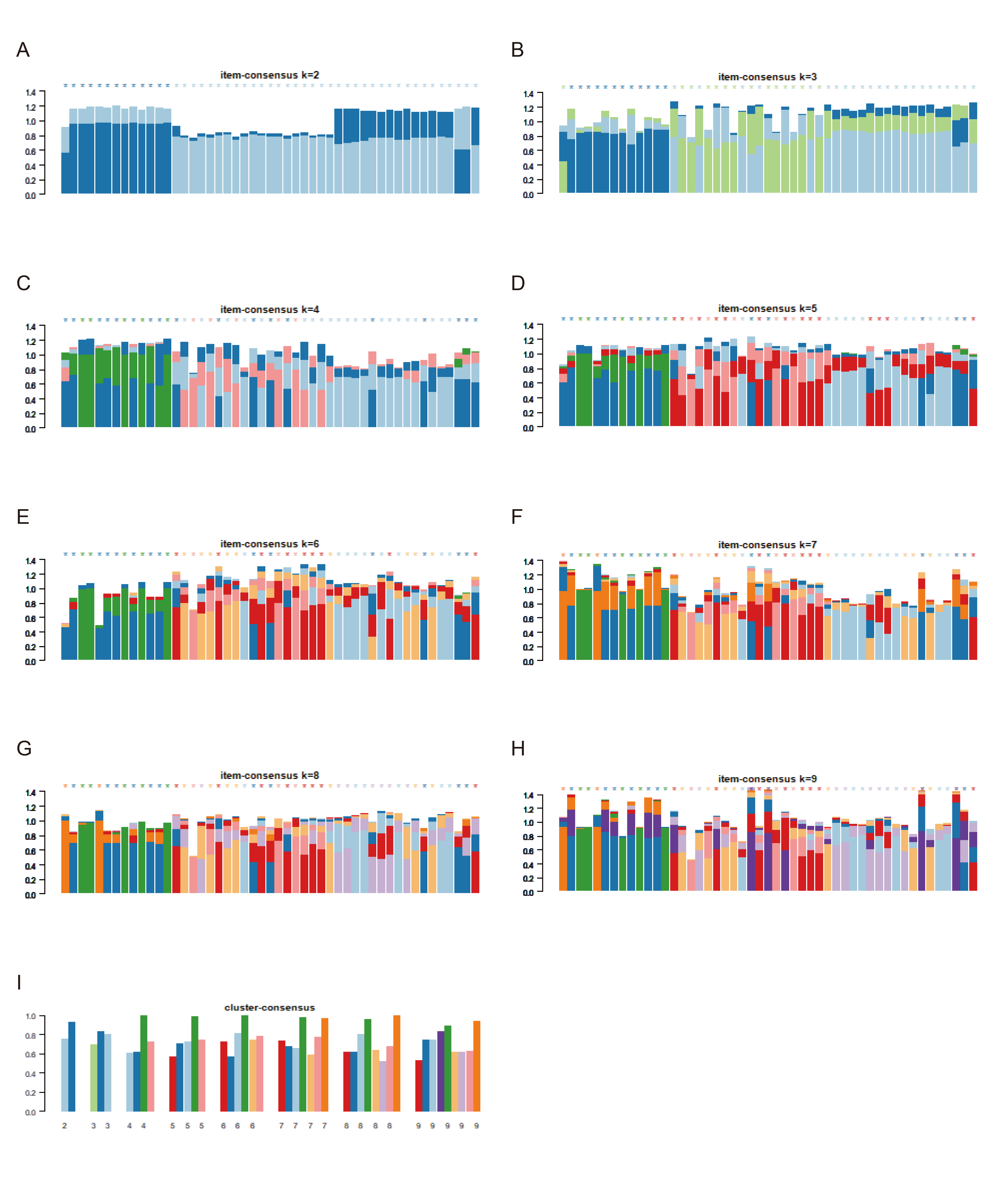


**Supplementary Figure S4.** The item and score of consensus clustering when k = 2-9. (A) The item of consensus clustering when k = 2-4. (B) The item of consensus clustering when k = 5-7. (C) The item of consensus clustering when k = 8-9. (D) The score of consensus clustering when k = 2-9.


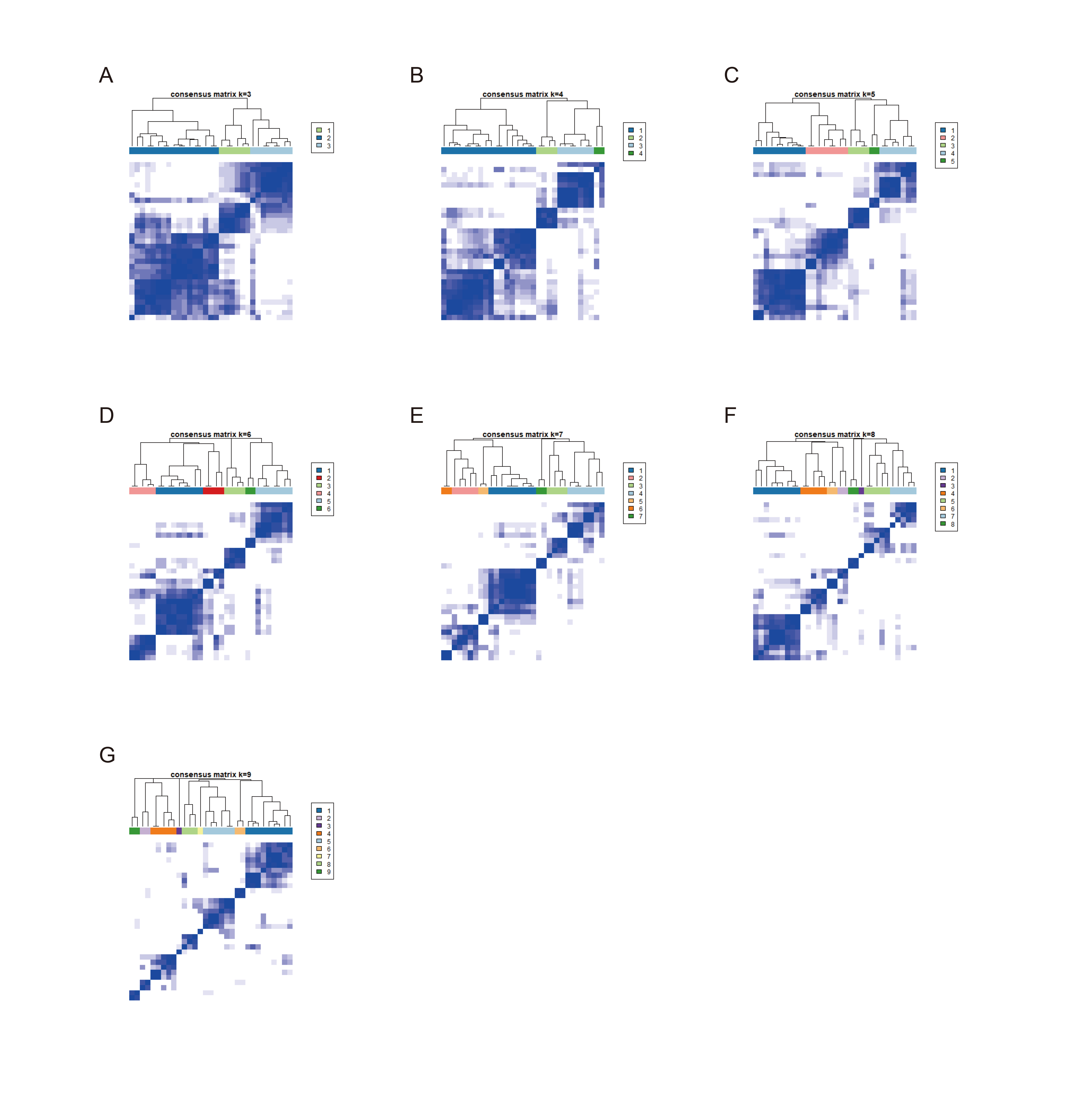


**Supplementary Figure S5.** The consensus clustering matrix when k = 3-9 in the validation set. (A) k = 3. (B) k = 4. (C) k = 5. (D) k = 6. (E) k = 7. (F) k = 8. (G) k = 9.


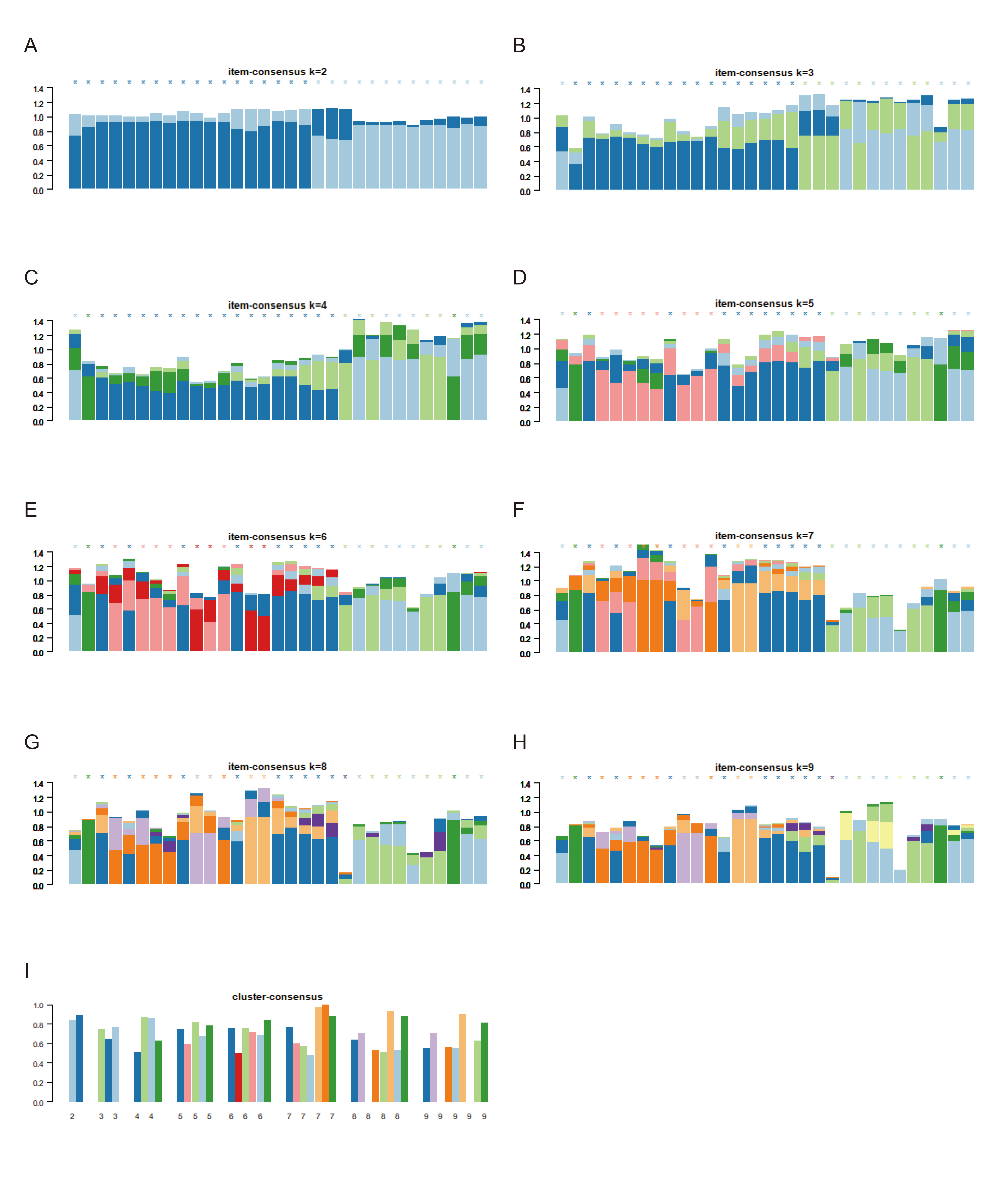


**Supplementary Figure S6.** The item and score of consensus clustering when k = 2-9 in the validation set. (A) The item of consensus clustering when k = 2-4. (B) The item of consensus clustering when k = 5-7. (C) The item of consensus clustering when k = 8-9. (D) The score of consensus clustering when k = 2-9.


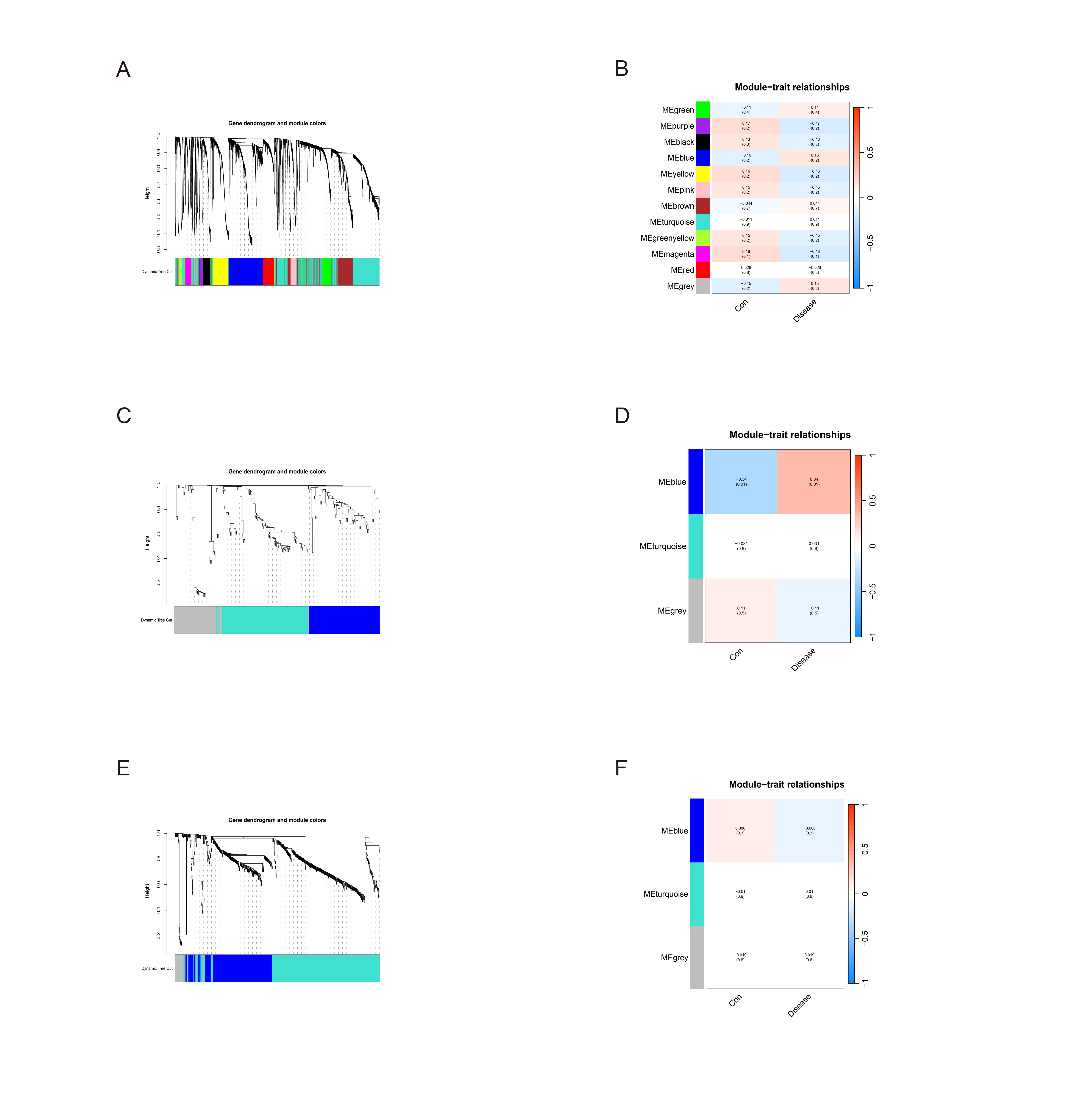


**Supplementary Figure S7.** Construction of WGCNA in datasets GSE19738, GSE48060 and GSE98793 (The cluster dendrogram and module-trait heatmap could not be obtained in the GSE34198 dataset using the WGCNA). (A) The cluster dendrogram of co-expression genes in the GSE19738 dataset. (B) The module-trait relationship heatmap in the GSE19738 dataset. (C) The cluster dendrogram of co-expression genes in the GSE48060 dataset. (D) The module-trait relationship heatmap in the GSE48060 dataset. (E) The cluster dendrogram of co-expression genes in the GSE98793 dataset. (F) The module-trait relationship heatmap in the GSE98793 dataset.

**The codes of data correction:**

qx=as.numeric(quantile(rt, c(0, 0.25, 0.5, 0.75, 0.99, 1.0), na.rm=T))

LogC=( (qx[5]>100) || ( (qx[6]-qx[1])>50 && qx[2]>0) )

if(LogC){

rt[rt<0]=0

rt=log2(rt+1)}

data=normalizeBetweenArrays(rt)
